# Comparison of the Safety and Efficacy of Remimazolam and Propofol for Sedation in Adults Undergoing Colonoscopy: A Meta-Analysis of Randomized Controlled Trials

**DOI:** 10.3390/medicina61040646

**Published:** 2025-04-01

**Authors:** Bon-Wook Koo, Hyo-Seok Na, Sang-Hi Park, Seunguk Bang, Hyun-Jung Shin

**Affiliations:** 1 Department of Anesthesiology and Pain Medicine, Seoul National University Bundang Hospital, Seongnam 13620, Republic of Korea; 2 Department of Anesthesiology and Pain Medicine, Seoul National University College of Medicine, Seoul 03080, Republic of Korea; 3 Department of Anesthesiology and Pain Medicine, Chungbuk National University Hospital, Cheongju-si 28644, Republic of Korea; 4 Department of Anesthesiology and Pain Medicine, Chungbuk National University College of Medicine, Cheongju-si 28644, Republic of Korea; 5 Department of Anesthesiology and Pain Medicine, Daejeon St. Mary’s Hospital, The Catholic University of Korea College of Medicine, Daejeon 34943, Republic of Korea; seungukb@naver.com

**Keywords:** remimazolam, propofol, colonoscopy, sedation, hemodynamics, respiratory insufficiency

## Abstract

*Background and Objectives*: This meta-analysis evaluates the safety and efficacy of remimazolam versus propofol for sedation during colonoscopy, focusing on hemodynamic and respiratory outcomes. *Materials and Methods*: A comprehensive search of CENTRAL, Embase, PubMed, Scopus, and Web of Science up to January 2025 identified randomized controlled trials (RCTs). Outcomes included hypotension (primary outcome), bradycardia, respiratory depression, injection pain, sedation onset time, emergence time, procedure success rate, and recovery room stay. Effect sizes were reported as relative risks (RR) or mean differences (MD) using random-effects models. *Results*: Fourteen RCTs with 3290 participants were included. Remimazolam significantly reduced the risk of hypotension (RR: 0.44, 95% CI [0.39, 0.51], *p* = 0.0000), bradycardia (RR: 0.36, 95% CI [0.25, 0.53], *p* = 0.0000), respiratory depression (RR: 0.32, 95% CI [0.22, 0.45], *p* = 0.0000), and injection pain (RR: 0.14, 95% CI [0.09, 0.24], *p* = 0.0000) compared to propofol. Remimazolam had slower sedation onset (MD: 15.97 s, 95% CI [8.30, 23.64], *p* = 0.0000) but allowed faster emergence (MD: −0.91 min, 95% CI [−1.69, −0.13], *p* = 0.023) and shorter recovery room stays (MD: −2.20 min, 95% CI [−3.23, −1.17], *p* = 0.0000). Both drugs had similar procedure success rates. *Conclusions*: Remimazolam demonstrates superior safety and efficacy compared to propofol, reducing risks of hypotension, bradycardia, respiratory depression, and injection pain while enabling faster recovery. These findings support remimazolam as a viable sedative for colonoscopy, though further large-scale studies are needed to confirm these results.

## 1. Introduction

Colonoscopy is a critical procedure for diagnosing and managing various gastrointestinal conditions, including colorectal cancer, which remains a leading cause of cancer-related mortality worldwide [1]. Effective sedation during colonoscopy is essential to ensure patient comfort, minimize procedural pain, and enhance overall diagnostic accuracy [2]. Traditionally, sedatives like propofol have been widely used due to their rapid onset and predictable recovery profiles. However, propofol use is often associated with adverse effects, including hypotension, bradycardia, and respiratory depression, which may compromise patient safety, particularly in vulnerable populations [3,4].

Remimazolam is a next-generation benzodiazepine designed for procedural sedation, offering a unique combination of rapid onset, short duration of action, and a favorable safety profile. Unlike traditional sedatives, it exerts minimal effects on cardiovascular and respiratory function, making it particularly well-suited for outpatient procedures and patients with underlying comorbidities. [5]. While propofol remains the widely used agent for sedation in colonoscopy, concerns about its narrow therapeutic window and cardiorespiratory suppression underscore the need for safer alternatives. However, direct comparative evidence between remimazolam and propofol has until recently been limited.

Pharmacologically, remimazolam undergoes rapid hydrolysis by nonspecific tissue esterases into an inactive metabolite, enabling organ-independent clearance and consistent recovery times—even with prolonged administration [5]. In contrast, propofol depends largely on hepatic metabolism and is associated with greater variability in clearance [6]. Additionally, the water-soluble nature of remimazolam eliminates the risk of injection-site pain, a common adverse effect of propofol, which is formulated in lipid emulsions [5,6]. These pharmacokinetics and pharmacodynamics characteristics may confer clinical advantages in various procedural sedation settings.

Emerging evidence from recent randomized trials and meta-analyses published between 2022 and 2024 has broadened the understanding of the role of remimazolam beyond colonoscopy [7]. Studies involving procedures such as bronchoscopy [8] and endoscopic retrograde cholangiopancreatography [9] have demonstrated that remimazolam provides comparable sedation quality to propofol while offering superior or comparable safety in terms of hemodynamic and respiratory outcomes. These findings highlight its potential utility as a versatile sedative agent across diverse diagnostic and therapeutic endoscopic procedures.

Given the widespread reliance on propofol and the growing interest in remimazolam, a comprehensive comparison of these agents is crucial to inform clinical practice. The hypothesis of this meta-analysis is that remimazolam provides superior safety and comparable efficacy to propofol for sedation during colonoscopy. By synthesizing data from randomized controlled trials (RCTs), this study aims to evaluate the hemodynamic and respiratory outcomes of both agents and provide evidence-based recommendations to optimize sedation practices.

## 2. Materials and Methods

This meta-analysis was designed and conducted in accordance with the 2020 Preferred Reporting Items for Systematic Reviews and Meta-Analyses (PRISMA) guidelines. Prior to initiating the study, the protocol was registered with International Prospective Register of Systematic Reviews (PROSPERO) and was allocated the registration number ‘CRD42025645206’.

### 2.1. Eligible Criteria

The study selection process followed established PICOS criteria (Population, Intervention, Comparison, Outcomes, and Study design): P, adults undergoing colonoscopy with sedation; I, use of remimazolam; C, comparison to propofol; O, RCTs reporting at least one relevant outcome. Studies that failed to meet these criteria, including conference abstracts, protocols, reviews, editorials, observational studies, case reports, or retrospective cohort studies, were excluded from the analysis.

### 2.2. Search Strategy

Eligible RCTs were located through an extensive search of electronic databases, such as CENTRAL, Embase, PubMed, Scopus, and Web of Science, covering the period from database inception until 14 January 2025. No restrictions were applied regarding language, publication year, region, or journal. Search terms included “Remimazolam”, “Byfavo”, “Colonoscopy”, and “Colono*”, which were combined using Boolean operators (AND/OR) to optimize the retrieval of relevant studies across these databases. The full search strategy can be found in Supplementary Table S1.

### 2.3. Study Selection and Data Extraction

All the studies retrieved from the databases were consolidated, and two investigators (BWK and HJS) independently carried out the selection of eligible RCTs. The process included removing duplicate entries, screening titles and abstracts, and reviewing full-text articles of studies that appeared relevant. After a comprehensive evaluation of the full texts, the final set of RCTs for data synthesis was established. Any disagreements between the investigators were resolved by consulting with a third investigator (HSN).

From the selected RCTs, key variables were extracted, systematically organized, and recorded on a data sheet. The data gathered included publication year, authors, participant count, age, remimazolam and propofol doses for sedation induction and maintenance, and outcomes such as hypotension, bradycardia, respiratory depression, time to reach the desired sedation depth, emergence time, injection pain, procedure success rates, and post-procedural unit stay time. Median values with interquartile ranges were converted into means and standard deviations using Wan’s formula [10].

There were no missing data related to the primary outcome (incidence of hypotension) among the included studies. For secondary outcomes with occasional incomplete reporting, we examined available study protocols and contacted corresponding authors for clarification when necessary. As missing data were minimal and did not affect the primary analysis, no imputation was required. This approach helped ensure data completeness and reliability in the meta-analysis.

### 2.4. Outcome Measures

The primary outcome was the incidence of hypotension during sedation. Secondary outcomes included the occurrence of bradycardia, respiratory depression, time to reach the desired sedation depth, emergence time, injection pain, procedure success rates, and post-procedural unit stay time.

### 2.5. Assessment of Risk of Bias and Quality of Evidence

The risk of bias for each outcome variable was assessed independently by two investigators (BWK and HJS) using the updated Cochrane risk of bias tool for randomized trials (RoB 2) [11]. Discrepancies in judgment were resolved through discussion and where consensus could not be reached, a third senior reviewer (HSN) was consulted. This process ensured inter-rater reliability and consistency in RoB assessment. The evaluation concentrated on five primary domains: (1) bias from the randomization process, (2) bias due to deviations from the intended interventions, (3) bias arising from incomplete outcome data, (4) bias in outcome measurement, and (5) bias concerning the selection of reported results. Each domain was categorized as having “low risk”, “some concerns”, or “high risk” of bias.

The certainty of evidence for each outcome was assessed using the Grading of Recommendations, Assessment, Development, and Evaluation (GRADE) framework [8]. The certainty of evidence for each outcome was assessed independently by two reviewers (BWK and HJS), with any discrepancies resolved through discussion or consultation with a third reviewer (HSN). This approach takes into account five crucial factors: (1) the risk of bias in the included studies, (2) inconsistencies in results across studies, (3) the directness of the evidence in addressing the research question, (4) the precision of the effect estimates, and (5) the potential effect of publication bias on the conclusions.

### 2.6. Statistical Analysis

Effect sizes for each study were determined using Stata^®^ SE version 17.0 (Stata Corp., College Station, TX, USA) to assess the influence of the interventions. For outcomes with binary data, the effect size was expressed as relative risk (RR) with a 95% confidence interval (CI), whereas for continuous outcomes, the mean difference (MD) was used, also accompanied by a 95% CI. A random-effects model was applied to all analyses to account for inter-study variability, offering a more precise estimate of the effect size. A significance threshold of *p* < 0.05 was used.

To examine heterogeneity in the pooled effect sizes, Cochran’s Q test and the I^2^ statistic were utilized, where I^2^ quantifies the extent of variability attributable to true differences between studies, rather than random chance. Heterogeneity levels were categorized as: none (I^2^ < 25%), low (I^2^ = 26–49%), moderate (I^2^ = 50–74%), and high (I^2^ > 75%). A sensitivity analysis, leaving one study out at a time, was carried out to investigate whether excluding any individual study would substantially change the pooled effect size, helping to detect potential small-study effects. Consistent with Cochrane guidelines [12], publication bias was not assessed using funnel plots or Egger’s test when fewer than 10 studies were included for each outcome.

## 3. Results

### 3.1. Study Selection

**Figure 1:**
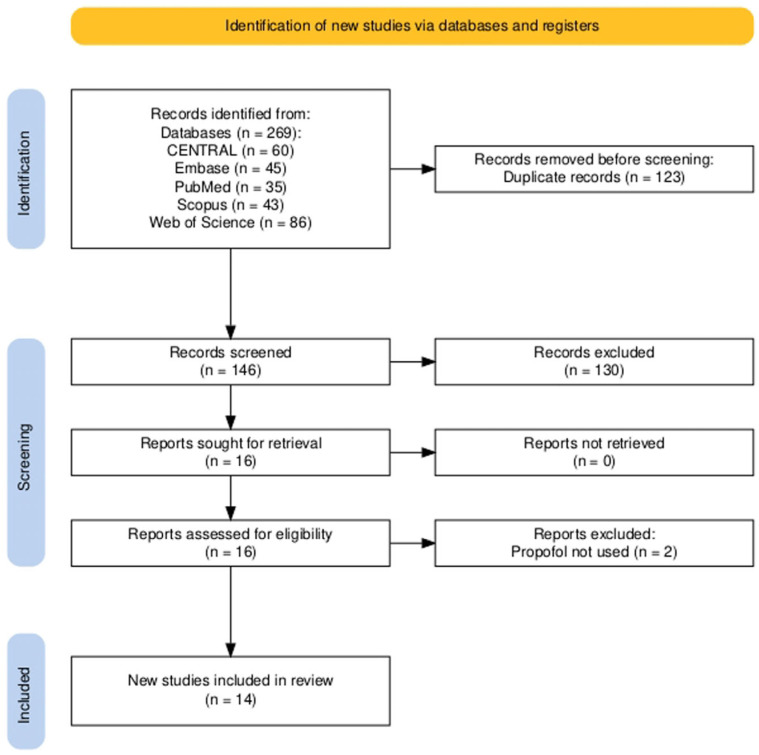
Flow diagram of study selection.

A total of 269 records were identified through searches of electronic databases. After eliminating 123 duplicate entries, the remaining records underwent a two-phase screening process. Initially, 102 studies were excluded based on their titles. Subsequently, a detailed review of the abstracts led to the exclusion of 28 additional studies. This process narrowed the selection to 16 studies for full-text review, of which 14 met the eligibility criteria and were included in the final meta-analysis. The complete selection process is illustrated in Figure 1.

### 3.2. Characteristics of Studies and Participants

Table 1 provides an overview of the key features of the RCTs included in the final analysis. In total, 3290 adults participated, with 1856 assigned to the remimazolam group and 1434 to the propofol group. The studies employed varying doses of remimazolam and propofol for both the induction and maintenance of sedation. In all studies, a predetermined dose was administered as a bolus for induction, while continuous infusion was used for maintaining sedation in three of the studies [13,14,15]. Definitions for outcomes such as hypotension, bradycardia, and respiratory depression are detailed in Supplementary Table S2.

### 3.3. Incidence of Hypotension

**Figure 2:**
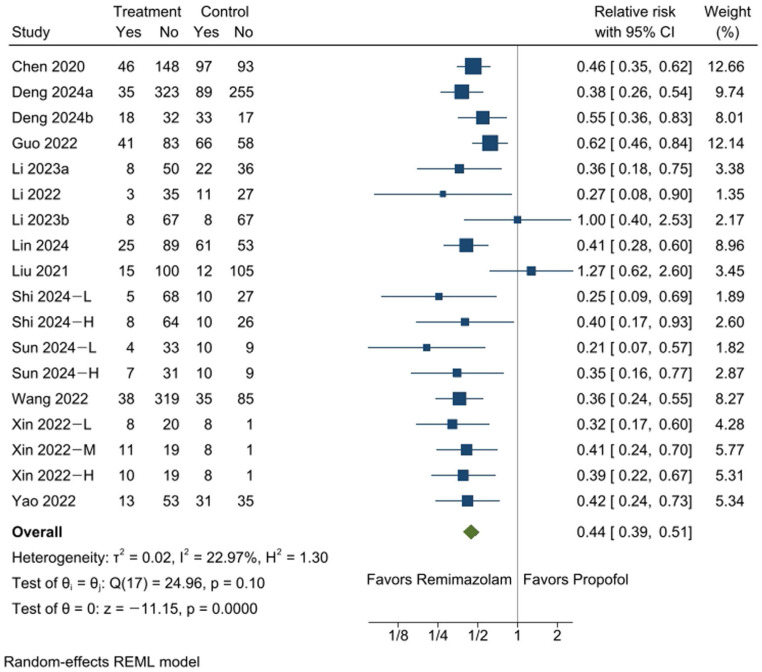
The forest plot illustrates the incidence of hypotension, showing a significantly lower occurrence in the remimazolam group compared to the propofol group. −L, −M, and −H indicate that the doses of remimazolam used in the study were relatively low, medium, and high, respectively. CI: confidence interval [13,14,15,16,17,18,19,20,21,22,23,24,25,26].

A total of 14 studies [13,14,15,16,17,18,19,20,21,22,23,24,25,26] encompassing 18 comparisons reported data on the incidence of hypotension. This condition was observed in 16.3% of participants in the remimazolam group (303 out of 1856) and 36.9% of those in the propofol group (529 out of 1434). The combined analysis demonstrated that remimazolam significantly reduced the likelihood of intraoperative hypotension during colonoscopy compared to propofol sedation (RR: 0.44, 95% CI [0.39, 0.51], *p* = 0.0000, I^2^ = 23%; Figure 2), corresponding to an absolute risk reduction (ARR) of 207 fewer events per 1000 patients, or 20.7% (95% CI: 22.5% to 18.1%) (Supplementary Table S3). This degree of reduction represents a substantial clinical benefit, particularly in patients with cardiovascular comorbidities or those susceptible to procedural hemodynamic instability. Therefore, in moderate- to high-risk settings, remimazolam may offer a safer alternative to propofol and potentially alter routine sedation practices.

A leave-one-out sensitivity analysis confirmed the stability of these findings, as no single study notably influenced the effect size (Supplementary Figure S1.1). While Egger’s test showed no statistical significance (*p* = 0.3246), potential publication bias was suggested by the absence of smaller studies in the nonsignificant region of the funnel plot (Supplementary Figure S1.2), as indicated by the trim-and-fill method (Supplementary Figure S1.3).

### 3.4. Incidence of Bradycardia

**Figure 3:**
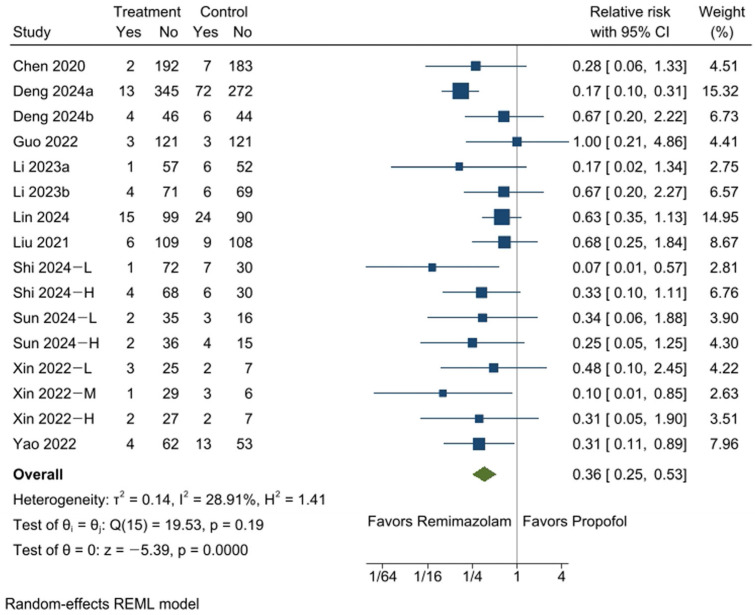
The forest plot presents the incidence of bradycardia during sedation, indicating a significantly lower rate in the remimazolam group compared to the propofol group. −L, −M, and −H indicate that the doses of remimazolam used in the study were relatively low, medium, and high, respectively. CI: confidence interval [13,15,16,17,18,19,20,21,22,23,25,26].

A total of 12 RCTs [13,15,16,17,18,19,20,21,22,23,25,26], comprising 16 comparisons, were analyzed to assess the incidence of bradycardia in the remimazolam and propofol groups. Bradycardia was observed less frequently in the remimazolam group, affecting 4.6% of participants (67 out of 1461), compared to 13.6% in the propofol group (173 out of 1276) (RR: 0.36, 95% CI [0.25, 0.53], *p* = 0.0000, I^2^ = 29%; Figure 3), with an ARR of 87 fewer per 1000, or 8.7% (95% CI: 10.2% to 6.4%) (Supplementary Table S3).

The sensitivity analysis demonstrated consistent pooled effect sizes, confirming the reliability of the findings (Supplementary Figure S2.1). Although Egger’s test showed no significant result (*p* = 0.4219), implying the absence of small-study effects or publication bias, asymmetry in the funnel plot was noted (Supplementary Figure S2.2), and adjustments were made using the trim-and-fill method (Supplementary Figure S2.3).

### 3.5. Incidence of Respiratory Depression

**Figure 4:**
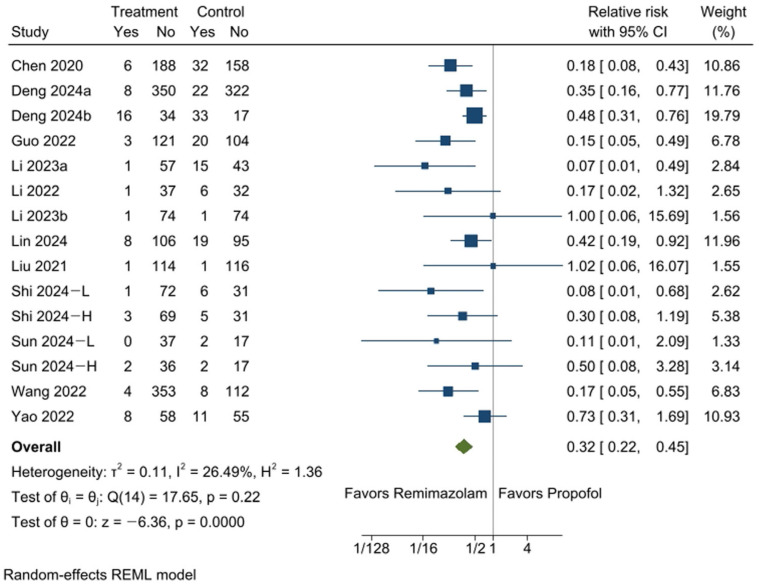
Forest plot for the incidence of respiratory depression during sedation. Participants in the remimazolam group showed a lower incidence of respiratory depression compared to those in the propofol group. −L and −H indicate that the doses of remimazolam used in the study were relatively low and high, respectively. CI: confidence interval [13,14,15,16,17,18,19,20,21,22,23,24,26].

Respiratory depression during sedation was evaluated in thirteen RCTs [13,14,15,16,17,18,19,20,21,22,23,24,26] with a total of fifteen comparisons. It occurred in 3.6% of participants in the remimazolam group (63 out of 1769) and 13.0% in the propofol group (183 out of 1407). The pooled analysis demonstrated that remimazolam significantly reduced the risk of intraoperative respiratory depression during sedation (RR: 0.32, 95% CI [0.22, 0.45], *p* = 0.0000, I^2^ = 27%; Figure 4). The ARR was 88 fewer per 1000, or 8.8% (95% CI: 10.1% to 7.2%) (Supplementary Table S3). While this reduction may not be practice-changing in healthy individuals, it is likely to be clinically meaningful in high-risk patients, such as those with respiratory compromise or obstructive sleep apnea. In such populations, the improved respiratory safety profile of remimazolam may influence sedation strategy selection.

Sensitivity analysis showed that removing any individual study did not meaningfully impact the pooled relative risk, affirming the robustness and reliability of the results (Supplementary Figure S3.1). While Egger’s test indicated no statistical significance (*p* = 0.1005), suggesting no strong evidence of small-study effects or publication bias, the funnel plot appeared asymmetric (Supplementary Figure S3.2), and adjustments were performed using the trim-and-fill method (Supplementary Figure S3.3).

### 3.6. Incidence of Injection Pain

The incidence of drug injection pain was analyzed in 12 RCTs [13,14,15,16,17,18,19,20,21,23,24,26], encompassing 13 comparisons. A notable difference was found between the remimazolam group, where 3.8% of participants (61 out of 1626) experienced pain, and the propofol group, with an incidence of 31.6% (421 out of 1334) (RR: 0.14, 95% CI [0.09, 0.24], *p* = 0.0000, I^2^ = 64%; Table 2). This corresponds to an absolute risk reduction of 271 events per 1000 patients, or 27.1% (95% CI: 28.7% to 24.0%) (Supplementary Table S3).

The leave-one-out sensitivity analysis confirmed that excluding any single study did not affect the pooled effect size for the incidence of pain (Supplementary Figure S4.1). However, the funnel plot showed asymmetry (Supplementary Figure S4.2), indicating potential publication bias. Egger’s test further supported this finding with statistically significant results (*p* = 0.0021). Additionally, the trim-and-fill method identified missing studies and imputed them accordingly (Supplementary Figure S4.3).

### 3.7. Procedure Success Rates

Nine studies [13,14,15,16,17,21,22,23,25], including thirteen comparisons, assessed procedure success rates. The pooled success rate in the remimazolam group was 97.4% (1122 out of 1152 participants), compared to 99.6% in the propofol group (959 out of 963 participants), showing no statistically significant difference between the groups (RR: 0.99, 95% CI [0.97, 1.00], *p* = 0.0958, I^2^ = 32%; Table 2). This represents an absolute risk reduction (ARR) of 1.0%, equivalent to 10 fewer events per 1000 patients (95% CI: 3.0% to 0%) (Supplementary Table S3).

Sensitivity analysis using the leave-one-out method revealed that the significance of the effect size for success incidence was influenced by the exclusion of a specific study [17], indicating its impact on the results (Supplementary Figure S5.1). The funnel plot appeared symmetrical (Supplementary Figure S5.2), and Egger’s test showed no significant findings (*p* = 0.5335). Additionally, the trim-and-fill method did not identify any missing studies (Supplementary Figure S5.3), suggesting no evidence of publication bias.

### 3.8. Time to Target Sedation Depth

Eleven RCTs [13,15,16,17,18,19,20,21,22,24,26] involving twelve comparisons were included in the analysis of the time required to achieve the target sedation depth in the remimazolam and propofol groups. Participants in the remimazolam group took longer to reach the desired sedation level compared to the propofol group (MD: 15.97 s, 95% CI [8.30, 23.64], *p* = 0.0000, I^2^ = 99%; Table 2).

The leave-one-out sensitivity analysis confirmed that the significance of the effect size remained stable regardless of which study was excluded (Supplementary Figure S6.1). Egger’s test revealed significant findings (*p* = 0.0425), the funnel plot appeared asymmetrical (Supplementary Figure S6.2), and the trim-and-fill method identified missing studies (Supplementary Figure S6.3), suggesting evidence of potential publication bias.

### 3.9. Emergence Time from Sedation

Emergence time from sedation was assessed in fourteen RCTs [13,14,15,16,17,18,19,20,21,22,23,24,25,26] across eighteen comparisons, revealing a notable difference between the remimazolam and propofol groups (MD: −0.91 min, 95% CI [−1.69, −0.31], *p* = 0.0230, I^2^ = 97%; Table 2).

The robustness of the findings was confirmed through a leave-one-out sensitivity analysis, which showed no significant changes in the effect size when any individual study was excluded (Supplementary Figure S7.1). Additionally, Egger’s test indicated no statistical significance (*p* = 0.6054), the funnel plot was symmetric (Supplementary Figure S7.2), and the trim-and-fill method (Supplementary Figure S7.3) did not detect any missing studies, suggesting an absence of publication bias.

### 3.10. Post-Procedural Unit Stay Time

Stay time in the post-procedural unit was assessed in 13 trials [13,14,15,16,17,18,19,20,21,22,23,24,25] (17 comparisons), and the pooled analysis demonstrated that stay time was significantly shorter in the remimazolam group compared to the propofol group (MD: −2.20 min, 95% CI [−3.23, −1.17], *p* = 0.0000, I^2^ = 95%; Table 2).

Sensitivity analysis using the leave-one-out method showed no changes in the effect size when individual studies were excluded (Supplementary Figure S8.1). No publication bias was detected through Egger’s test, the funnel plot (Supplementary Figure S8.2), and the trim-and-fill method (Supplementary Figure S8.3).

### 3.11. Risk of Bias

Supplementary Figures S9.1 and S9.2 summarize the overall assessment of bias risk. The majority of studies analyzed in this meta-analysis were classified as having a “low” risk of bias [13,14,15,16,17,18,19,20,21,22,24,25,26]. However, one study [23] raised “some concerns” due to insufficient details regarding the concealment of group allocation prior to participant enrollment and intervention.

### 3.12. Certainty of Evidence

The evaluation of evidence certainty is detailed in Supplementary Table S3. Six outcomes—hypotension, bradycardia, respiratory depression, injection pain, procedure success rate, and time to reach the target sedation depth—were classified as having moderate certainty, largely due to concerns about potential bias. In contrast, other outcomes, such as emergence time from sedation and duration of stay in the post-procedure care unit, were rated with low certainty, attributed to substantial heterogeneity and notable bias-related concerns.

## 4. Discussion

The findings of the present meta-analysis provide strong and compelling evidence to support the notion that remimazolam represents a safe and effective alternative to propofol for sedation during colonoscopy. In comparison to propofol, remimazolam significantly lowered the incidence of adverse events, including hypotension, bradycardia, respiratory depression, and injection-related pain. These results suggest that remimazolam has the potential to improve patient safety, particularly in populations at higher risk of hemodynamic instability or respiratory compromise during procedural sedation.

The remarkable hemodynamic safety profile, demonstrated by a reduction in the risks of hypotension (56%) and bradycardia (64%)—both commonly observed with propofol use [3]—is a key strength of remimazolam. This suggests that remimazolam may be especially advantageous for patients with existing cardiovascular vulnerabilities or those undergoing procedures in settings where maintaining hemodynamic stability is critically important. By mitigating the risk of significant changes such as drops in blood pressure and heart rate—two valuable markers of cardiovascular health—remimazolam helps reduce the likelihood of adverse cardiovascular events during sedation, thereby enhancing overall procedural safety.

In addition to its hemodynamic benefits, remimazolam also demonstrated a significantly lower risk of respiratory depression, a common and concerning complication associated with sedative agents such as propofol [4]. This advantage makes remimazolam a valuable option for patients with pre-existing respiratory concerns, such as those with chronic obstructive pulmonary disease, obstructive sleep apnea, or other conditions that predispose them to respiratory compromise. The minimal respiratory depression observed with remimazolam may decrease the requirement for management such as supplemental oxygen or invasive airway support during the procedure, contributing to a safer sedation experience.

In terms of sedation efficacy, remimazolam showed several advantages over propofol, including faster emergence times and shorter stays in the recovery room. These factors represent its ability to facilitate quicker patient recovery, allowing for more efficient turnover in high-volume procedural settings like ambulatory surgical centers or outpatient clinics. Although the slower sedation onset of remimazolam than propofol (about 16 s) may be a drawback, this is effectively counterbalanced by its rapid emergence from sedation, which contributes to reduced recovery times and overall enhanced procedural efficiency.

Our results align with prior meta-analyses that support favorable safety profile of remimazolam [7]. However, combination sedation regimens such as propofol-midazolam or propofol-ketamine remain widely used due to their synergistic effects on sedation depth and onset time. Therefore, role of remimazolam in sedation strategies should be further evaluated in comparison with these commonly used regimens.

From a clinical perspective, remimazolam may be an alternative to propofol, particularly for populations who are at elevated risk for cardiovascular or respiratory complications. The pharmacokinetic profile of remimazolam, which includes organ-independent metabolism and minimal accumulation in the body [5], further contributes to its interest. These characteristics allow remimazolam to be a versatile option for use across diverse patient populations, including those with complex medical histories or comorbid conditions. Additionally, the reduced need for adjunctive medications to manage adverse effects means that sedation protocols can be simplified, potentially reducing the complexity of care associated with procedural sedation.

Although our findings favor remimazolam in terms of safety outcomes, certain limitations should be considered. Remimazolam has a slower onset of sedation compared to propofol (MD: 15.97 s), which may be a disadvantage in high-throughput procedural environments where rapid induction is prioritized. Moreover, the higher cost of remimazolam and its limited availability in some healthcare systems may restrict its routine use, particularly in settings where propofol is already well-established and cost-effective.

It is important to note that the interpretation of these findings should consider several limitations. The heterogeneity among the studies included in this meta-analysis, particularly with regard to differences in sedation protocols, dosing regimens, and patient populations, may have influenced the outcomes. While the use of a random-effects model helped to account for some of this variability, high statistical heterogeneity was still observed for certain outcomes, such as time to target sedation and emergence time. This highlights the potential impact of study design on the results and suggests that the findings should be interpreted with caution. Furthermore, evidence of publication bias for some endpoints, such as injection pain, underscores the need for a careful assessment of the available literature. Additionally, many of the studies included in this meta-analysis focused primarily on short-term perioperative outcomes, with limited data on long-term safety, patient satisfaction, and cost-effectiveness. Future research should seek to address these gaps, particularly with respect to the long-term effects of remimazolam use in procedural sedation.

Moreover, the applicability of these results in resource-limited settings or in populations with complex comorbidities remains an important area for future investigation. Further studies are needed to explore how remimazolam performs in these settings, as well as its potential to reduce the burden of sedation-related complications in diverse patient groups.

## 5. Conclusions

In conclusion, this meta-analysis provides robust evidence that remimazolam has the potential to be one of the sedative practices in colonoscopy by offering a safe and effective alternative to propofol. The drug’s favorable safety profile, which includes reduced risks of hypotension, bradycardia, respiratory depression, and injection pain, positions it as a valuable tool for improving patient safety during sedation. Additionally, its shorter recovery times and fast emergence from sedation make it a useful option in high-turnover procedural environments. Future research should also include randomized controlled trials comparing remimazolam not only with propofol alone but also with widely used multi-agent sedation protocols, such as propofol–midazolam or propofol–ketamine, to determine the optimal strategy for balancing efficacy, safety, and efficiency. Furthermore, large-scale, multicenter real-world studies are needed to evaluate the cost-effectiveness, long-term safety, and implementation feasibility of remimazolam across diverse healthcare settings. These efforts will be critical for guiding clinical practice and informing sedation guidelines.

## Figures and Tables

**Table 1:** Characteristics of the studies included.

Author (Year)	No. of Group	Sample Size		Age		Induction		Maintenance		Opioid
		N1	N2	A1	A2	Remimazolam	Propofol	Remimazolam	Propofol	
Chen, 2020 [16]	2	194	190	45	44	5 mg	1.5 mg/kg	2.5 mg	0.5 mg/kg	Fentanyl 1 µg/kg
Deng, 2024a [17]	2	358	344	75	75	0.15 mg/kg	1.5 mg/kg	0.05 mg/kg	0.5 mg/kg	Esketamine 0.3 mg/kg
Deng, 2024b [18]	2	50	50	46	47	0.15 mg/kg	2 mg/kg	2.5 mg	0.5 mg/kg	Sufentanil 0.1 µg/kg
Guo, 2022 [19]	2	124	124	47	48	5 mg	1.5 mg/kg	2.5 mg	0.5 mg/kg	Fentanyl 1 µg/kg
Li, 2023a [13]	2	58	58	49	48	0.05–0.15 mg/kg	0.5–1.5 mg/kg	0.3–0.5 mg/kg/h	3–5 mg/kg/h	Sufentanil 0.1 µg/kg
Li, 2022 [14]	2	38	38	68	68	0.05–0.15 mg/kg	0.5–1.5 mg/kg	0.3–0.5 mg/kg/h	3–5 mg/kg/h	Sufentanil 0.1 µg/kg
Li, 2023b [15]	2	75	75	50	50	0.15–0.25 mg/kg	1–2 mg/kg	1–2 mg/kg/h	2–4 mg/kg/h	Esketamine 0.15 mg/kg
Lin, 2024 [20]	2	114	114	71	71	0.2 mg/kg	1 mg/kg	0.1 mg/kg	0.5 mg/kg	Sufentanil 0.05 µg/kg
Liu, 2021 [21]	2	115	117	69	69	0.15 mg/kg	0.1 mL/kg	0.075 mg/kg	0.05 mL/kg	Fentanyl 0.5 µg/kg
Shi, 2024 [22]	3	73	73	58	60	0.15 mg/kg	2 mg/kg	0.04 mg/kg	0.4 mg/kg	Nalbuphine 0.2 mg/kg
		72		57		2 mg/kg	2 mg/kg	0.04 mg/kg	0.4 mg/kg	Nalbuphine 0.2 mg/kg
Sun, 2024 [23]	3	37	38	53	53	0.2 mg/kg	2 mg/kg	2.5 mg	30 mg	Sufentanil 5 µg
		38		48		0.25 mg/kg	2 mg/kg	2.5 mg	30 mg	Sufentanil 5 µg
Wang, 2022 [24]	2	357	120	44	47	7 mg/kg	1.5 mg/kg	2.5 mg	0.5 mg/kg	Fentanyl 50 µg
Xin, 2022 [25]	4	28	27	54	56	0.1 mg/kg	2 mg/kg	2.5 mg	1/3 to 1/2 of initial dose	Alfentanil 10 µg/kg
		30		50		0.15 mg/kg	2 mg/kg	2.5 mg	1/3 to 1/2 of initial dose	Alfentanil 10 µg/kg
		29		52		0.2 mg/kg	2 mg/kg	2.5 mg	1/3 to 1/2 of initial dose	Alfentanil 10 µg/kg
Yao, 2022 [26]	3	66	66	49	48	0.2 mg/kg	1 mg/kg	6 mg	30 mg	Sufentanil 5 µg

A1: mean age in remimazolam group, A2: mean age in the propofol group, N1: number of participants in the remimazolam group, N2: number of participants in the propofol group.

**Table 2:** Summary of pooled effect sizes for sedation-related outcomes.

	No. of Studies	No. of Participants	RR (95% CI)	*p* Value	I^2^
Injection pain	12 (13 comparisons)	2960	0.14 [0.09, 0.24]	0.0000	64%
Procedure success rates	9 (13 comparisons)	2115	0.99 [0.97, 1.00]	0.0958	32%
	No. of studies	No. of participants	MD (95% CI)	*p* value	I^2^
Time to target sedation depth	11 (12 comparisons)	1801	15.97 [8.30, 23.64]	0.0000	99%
Emergence time from sedation	14 (18 comparisons)	3290	−0.91 [−1.69, −0.31]	0.0230	97%
Post-procedural unit stay time	13 (17 comparisons)	3158	−2.20 [−3.23, −1.17]	0.0981	95%

RR: relative risk, MD: mean difference, CI: confidence interval.

## Data Availability

The original contributions presented in this study are included in the article/Supplementary Materials. Further inquiries can be directed to the corresponding author.

## Supplementary Materials

The following supporting information can be downloaded at: https://www.mdpi.com/article/10.3390/medicina61040646/s1. Table S1: Search strategy for each database 20250114. Table S2: Definition of hemodynamic variables. Table S3: Certainty for each outcome. Figure S1.1: Forest plot for sensitivity analysis of the incidence of hypotension in the comparison between remimazolam and propofol groups. Figure S1.2: Funnel plot for the incidence of hypotension. Figure S1.3: Funnel plot with trim-and-fill method for the incidence of hypotension. Figure S2.1: Forest plot for the sensitivity analysis of the incidence of bradycardia. Figure S2.2: Funnel plot for the incidence of bradycardia. Figure S2.3: Funnel plot with trim-and-fill method for the incidence of bradycardia. Figure S3.1: Forest plot for sensitivity analysis of incidence of respiratory depression. Figure S3.2: Funnel plot for the incidence of respiratory depression. Figure S3.3: Funnel plot with trim-and-fill method for the incidence of respiratory depression. Figure S4.1: Forest plot for sensitivity analysis of the incidence of injection pain. Figure S4.2: Funnel plot for the incidence of injection pain. Figure S4.3: Funnel plot with trim-and-fill method for the incidence of injection pain. Figure S5.1: Forest plot for sensitivity analysis of success rates. Figure S5.2: Funnel plot for the success rates. Figure S5.3: Funnel plot with trim-and-fill method for the success rates. Figure S6.1: Forest plot for sensitivity analysis of time to target sedation depth. Figure S6.2: Funnel plot for time to target sedation depth. Figure S6.3: Funnel plot with trim-and-fill method for time to target sedation depth. Figure S7.1: Forest plot for sensitivity analysis of emergence time from sedation. Figure S7.2: Funnel plot for emergence time from sedation. Figure S7.3: Funnel plot with trim-and-fill method for emergence time from sedation. Figure S8.1: Forest plot for sensitivity analysis of post-procedural unit stay time. Figure S8.2: Funnel plot for post-procedural unit stay time. Figure S8.3: Funnel plot with trim-and-fill method for post-procedural unit stay time. Figure S9.1: Risk of bias summary. Figure S9.2: Overall risk of bias as a summary plot.

## Abbreviations

The following abbreviations are used in this manuscript:

RCT	Randomized controlled trial
RR	Relative risk
MD	Mean difference
CI	Confidence interval
PK	Pharmacokinetics
PD	Pharmacodynamics
PRISMA	Preferred Reporting Items for Systematic Reviews and Meta-Analyses
PROSPERO	International Prospective Register of Systematic Reviews
RoB 2	Cochrane Risk of Bias tool 2
GRADE	Grading of Recommendations, Assessment, Development, and Evaluation
