# Supplementary material for: Comparison of the Safety and Efficacy of Remimazolam and Propofol for Sedation in Adults Undergoing Colonoscopy: A Meta-Analysis of Randomized Controlled Trials
Source: Medicina (Kaunas). 2025 Apr 1;61(4):646. doi: 10.3390/medicina61040646 (PMC12028776; doi:10.3390/medicina61040646)
Supplement: Supplementary file 1 [file medicina-61-00646-s001.zip › medicina-3541244-supplementary.pdf]

Supplementary Files

Supplementary Table S1. Search strategy for each database 20250114

Database	Order	Keywords	Results
PubMed	#1	"Remimazolam"[Title/Abstract] OR "Byfavo"[Title/Abstract]	786
	#2	"Colonoscopy"[Title/Abstract] OR "colono*"[Title/Abstract]	44,074
	#3	#1 AND #2	**35**
EMBASE	#1	remimazolam:ab,ti OR byfavo:ab,ti	816
	#2	colonoscopy:ab,ti OR colono*:ab,ti	86,955
	#3	#1 AND #2	**45**
CENTRAL	#1	(Remimazolam OR Byfavo):ti,ab,kw in trials	1,119
	#2	(Colonoscopy OR Colono*):ti,ab,kw in trials	9,858
	#3	#1 AND #2	**60**
SCOPUS	#1	TITLE-ABS-KEY (remimazolam OR byfavo)	958
	#2	TITLE-ABS-KEY (colonoscopy OR colono*)	98,739
	#3	#1 AND #2 AND (LIMIT-TO (DOCTYPE , "ar"))	**43**
Web of Science	#1	Remimazolam OR Byfavo (Topic)	821
	#2	Colonoscopy OR Colono* (Topic)	54,733
	#3	#1 AND #2 and Article (Document Types)	**86**

Supplementary Table S2. Definition of hemodynamic variables

Study	Definition
**Hypotension**	
Chen 2020	SBP ≤ 80 mmHg or a decrease of ≥ 20% from baseline
Deng 2024a	SBP < 90 mmHg or a DBP < 60 mmHg or a decrease of > 20% from baseline
Deng 2024b	A decrease of > 30% from baseline
Guo 2022	SBP or DBP decrease of > 20% from baseline
Li 2023a	SBP < 90 mmHg or a decrease of > 20% from baseline
Li 2022	SBP < 90 mmHg or a decrease of > 20% from baseline
Li 2023b	MBP decrease of > 20% from baseline
Lin 2024	MBP < 65 mmHg or ≥ 30% decrease from baseline
Liu 2021	SBP < 90 mmHg, DBP < 50 mmHg, or a MBP decrease of 20% or more below baseline
Shi 2024	SBP < 90 mmHg or a decrease of > 20% from baseline
Sun 2024	SBP < 90 mmHg or a SBP or DBP decrease of > 30% from baseline
Wang 2022	SBP ≤ 80 mmHg
Xin 2022	A decrease of > 20% from baseline
Yao 2022	MBP decrease of > 20% from baseline
**Bradycardia**	
Chen 2020	No definition
Deng 2024a	HR < 50 beats/minute or a decrease of > 20% from baseline
Deng 2024b	HR < 45 beats/minute
Guo 2022	No definition
Li 2023a	No definition
Li 2022	HR < 50 beats/minute
Li 2023b	HR < 50 beats/minute
Lin 2024	HR < 50 beats/minute
Liu 2021	HR < 50 beats/minute or a decrease of > 20% from baseline
Shi 2024	HR < 50 beats/minute
Sun 2024	HR < 50 beats/minute

Wang 2022	No definition
Xin 2022	HR < 60 beats/minute
Yao 2022	HR < 50 beats/minute
**Respiratory depression**	
Chen 2020	SpO_2 < 90% and or a respiratory rate of <8 breaths/min
Deng 2024a	SpO_2 < 90% and or a respiratory rate of <8 breaths/min
Deng 2024b	SpO_2 < 90%
Guo 2022	SpO_2 < 90% or a respiratory rate of <8 breaths/min
Li 2023a	SpO_2 < 90% or a respiratory rate of <8 breaths/min
Li 2022	SpO_2 < 90% or a respiratory rate of <8 breaths/min
Li 2023b	SpO_2 < 90% for more than 10 seconds
Lin 2024	SpO_2 < 90%
Liu 2021	SpO_2 < 90% and or a respiratory rate of <8 breaths/min
Shi 2024	SpO_2 < 90% for more than 1 minute
Sun 2024	SpO_2 < 92%
Wang 2022	SpO_2 < 90%
Xin 2022	SpO_2 < 90% for more than 20 seconds
Yao 2022	SpO_2 < 92%

Supplementary Table S3. Certainty for each outcome

Certainty assessment							№ of patients		Effect		Certainty	Importance
№ of studies	Study design	Risk of bias	Inconsistency	Indirectness	Imprecision	Other considerations	RMZ	PPF	Relative (95% CI)	Absolute (95% CI)		
**Hypotension**												
14	randomised trials	serious[a]	not serious	not serious	not serious	none	303/1856 (16.3%)	529/1434 (36.9%)	**RR 0.44** (0.39 to 0.51)	**207 fewer per 1,000** (from 225 fewer to 181 fewer)	⨁⨁⨁◯ Moderate[a]	
**Bradycardia**												
12	randomised trials	serious[a]	not serious	not serious	not serious	none	67/1461 (4.6%)	173/1276 (13.6%)	**RR 0.36** (0.25 to 0.53)	**87 fewer per 1,000** (from 102 fewer to 64 fewer)	⨁⨁⨁◯ Moderate[a]	
**Respiratory depression**												
13	randomised trials	serious[a]	not serious	not serious	not serious	none	63/1769 (3.6%)	183/1407 (13.0%)	**RR 0.32** (0.22 to 0.45)	**88 fewer per 1,000** (from 101 fewer to 72 fewer)	⨁⨁⨁◯ Moderate[a]	

Certainty assessment							№ of patients		Effect		Certainty	Importance
№ of studies	Study design	Risk of bias	Inconsistency	Indirectness	Imprecision	Other considerations	RMZ	PPF	Relative (95% CI)	Absolute (95% CI)		
**Injection pain**												
12	randomised trials	serious[a]	not serious	not serious	not serious	none	61/1626 (3.8%)	421/1334 (31.6%)	**RR 0.14** (0.09 to 0.24)	**271 fewer per 1,000** (from 287 fewer to 240 fewer)	⨁⨁⨁◯ Moderate[a]	
**Success rate**												
9	randomised trials	serious[a]	not serious	not serious	not serious	none	1122/1152 (97.4%)	959/963 (99.6%)	**RR 0.99** (0.97 to 1.00)	**10 fewer per 1,000** (from 30 fewer to 0 fewer)	⨁⨁⨁◯ Moderate[a]	
**Time to target sedatioin depth**												
11	randomised trials	not serious	serious[b]	not serious	not serious	none	1054	747	-	MD **15.97 seconds more** (8.3 more to 23.64 more)	⨁⨁⨁◯ Moderate[b]	
**Emergence time**												

Certainty assessment							№ of patients		Effect		Certainty	Importance
№ of studies	Study design	Risk of bias	Inconsistency	Indirectness	Imprecision	Other considerations	RMZ	PPF	Relative (95% CI)	Absolute (95% CI)		
14	randomised trials	serious[a]	serious[b]	not serious	not serious	none	1856	1434	-	MD **0.91 minutes fewer** (1.69 fewer to 0.13 fewer)	⨁⨁◯◯ Low[a,b]	

Post-procedural stay time

13	randomised trials	serious[a]	serious[b]	not serious	not serious	none	1790	1368	-	MD **2.2 minutes fewer** (3.23 fewer to 1.17 fewer)	⨁⨁◯◯ Low[a,b]	

CI: confidence interval; **MD:** mean difference; **RR:** risk ratio

Explanations

a. Downgrade for some concerns in risk of bias

b. Downgrade for inconsistency (High heterogeneity)

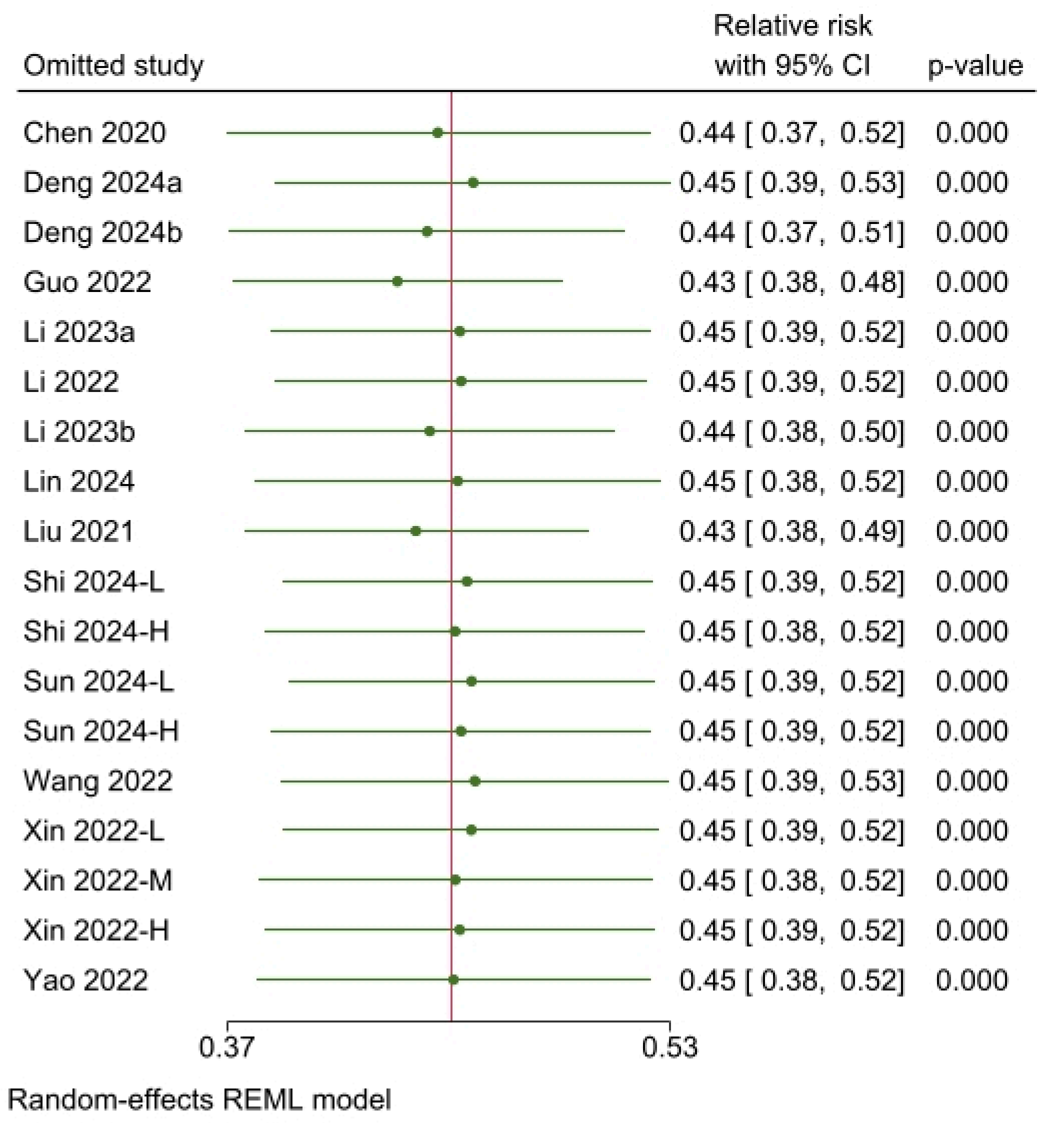

Supplementary Figure S1.1. Forest plot for sensitivity analysis of the incidence of hypotension in the comparison between remimazolam and propofol groups. Sensitivity analysis identified no alteration of effect size. CI: confidence interval.

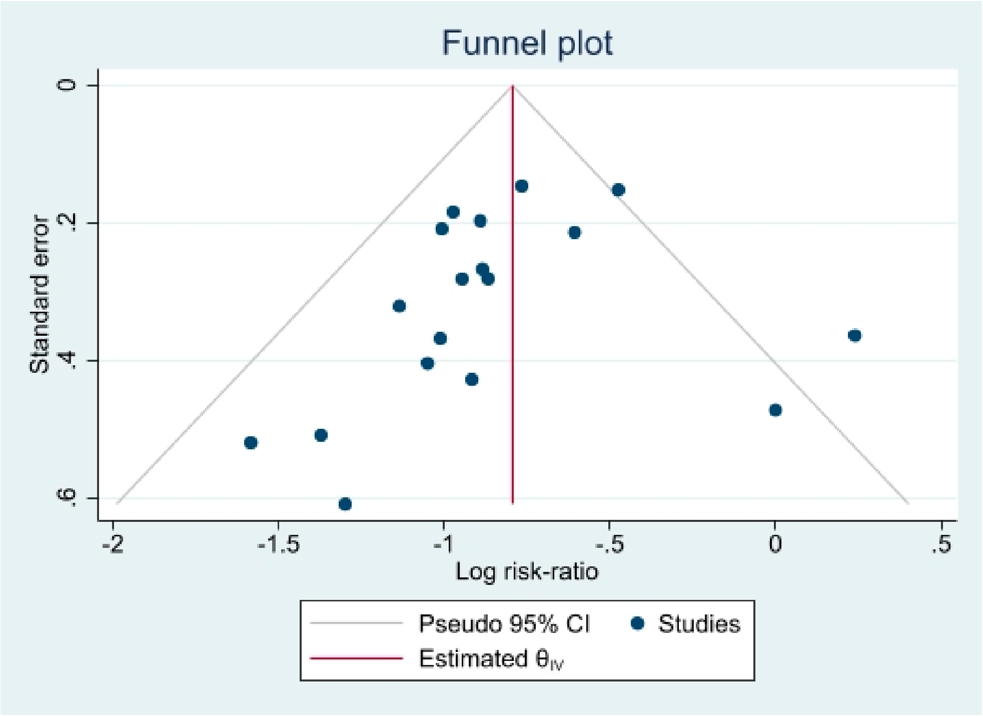

Supplementary Figure S1.2. Funnel plot for the incidence of hypotension. CI, confidence interval.

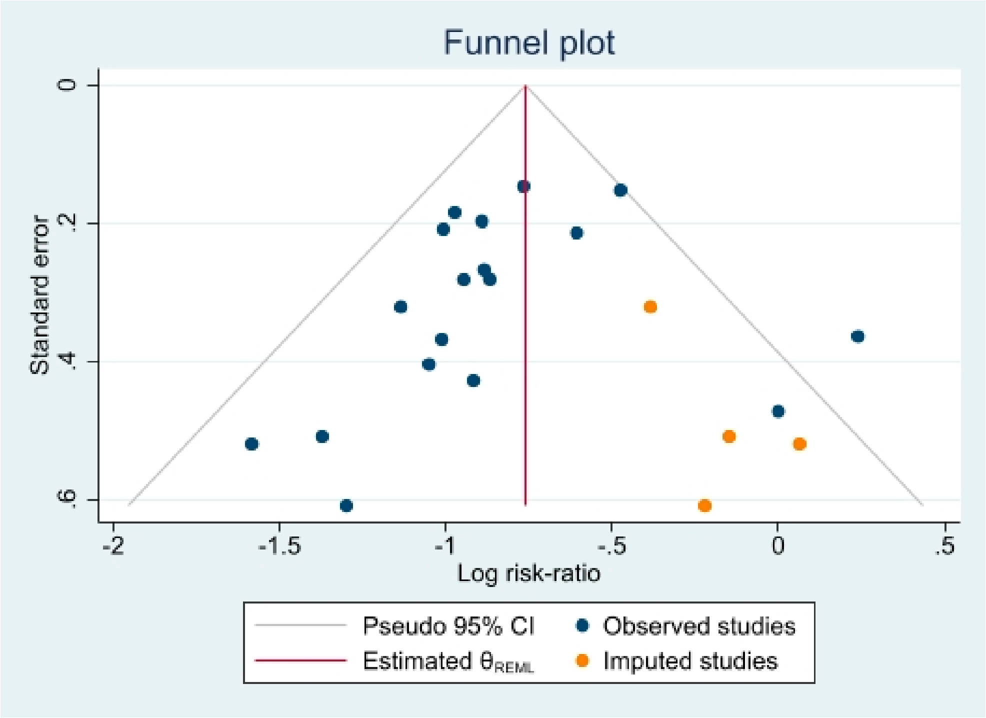

Supplementary Figure S1.3. Funnel plot with trim-and-fill method for the incidence of hypotension. CI, confidence interval

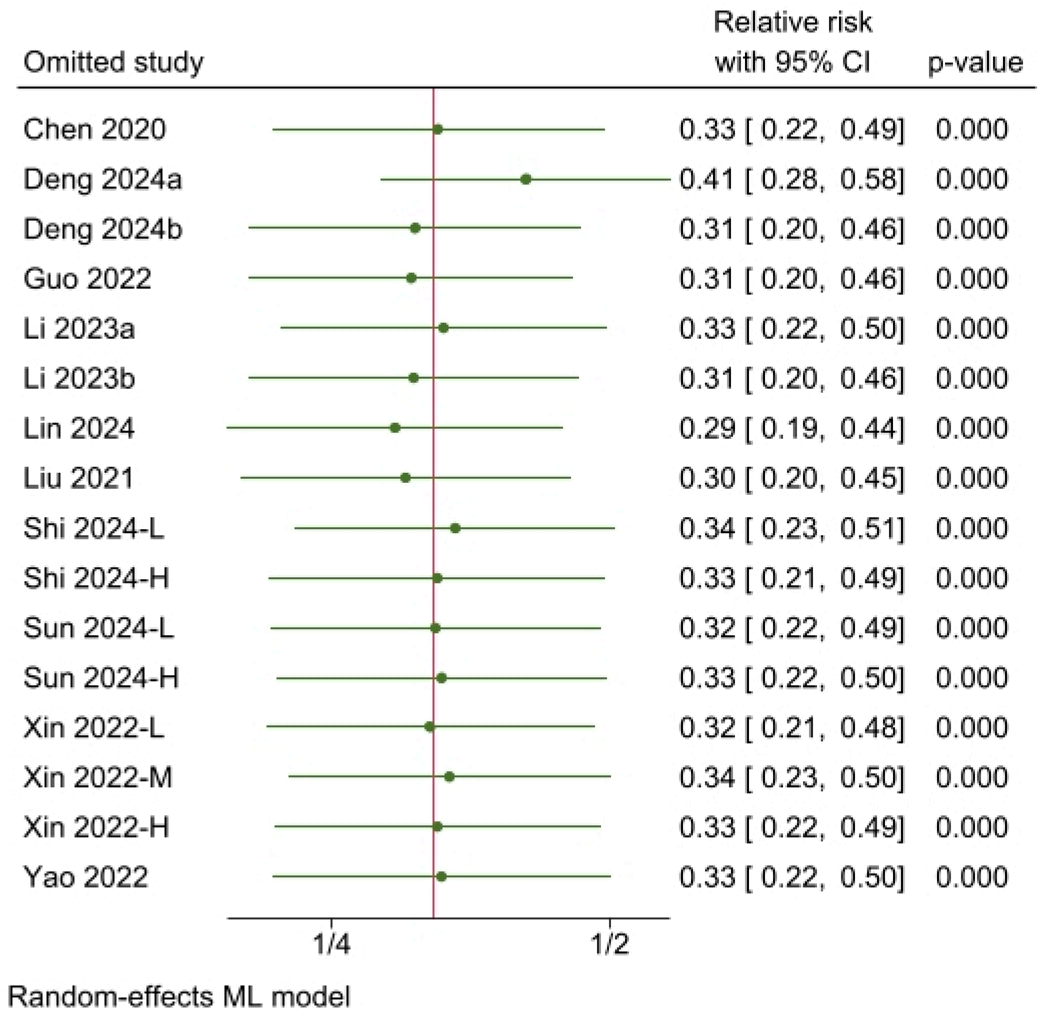

Supplementary Figure S2.1. Forest plot for the sensitivity analysis of the incidence of bradycardia comparing the remimazolam and propofol groups.

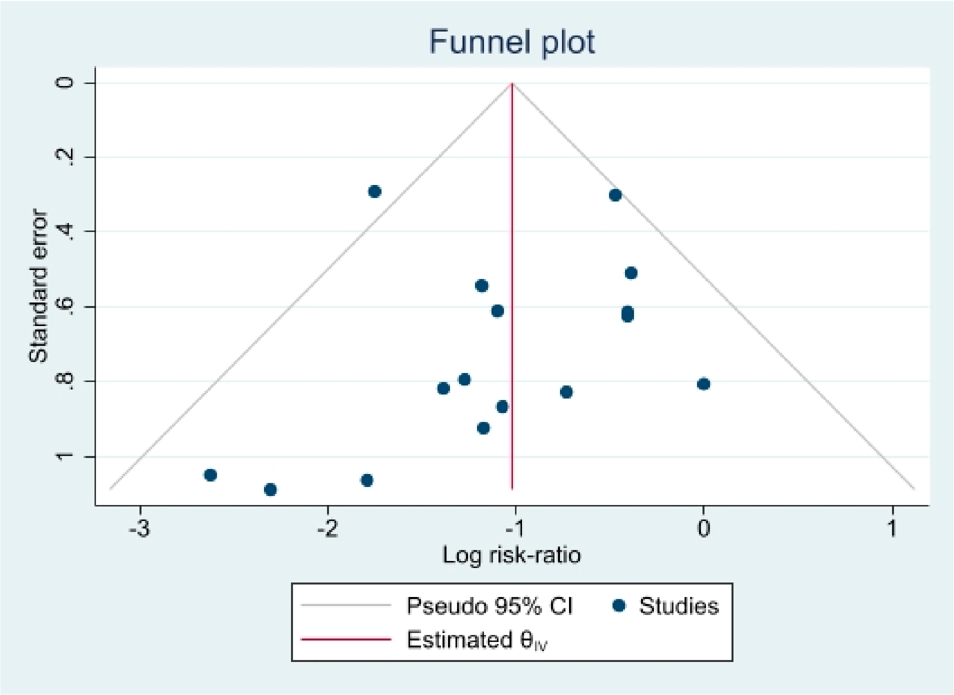

Supplementary Figure S2.2. Funnel plot for the incidence of bradycardia. CI, confidence interval.

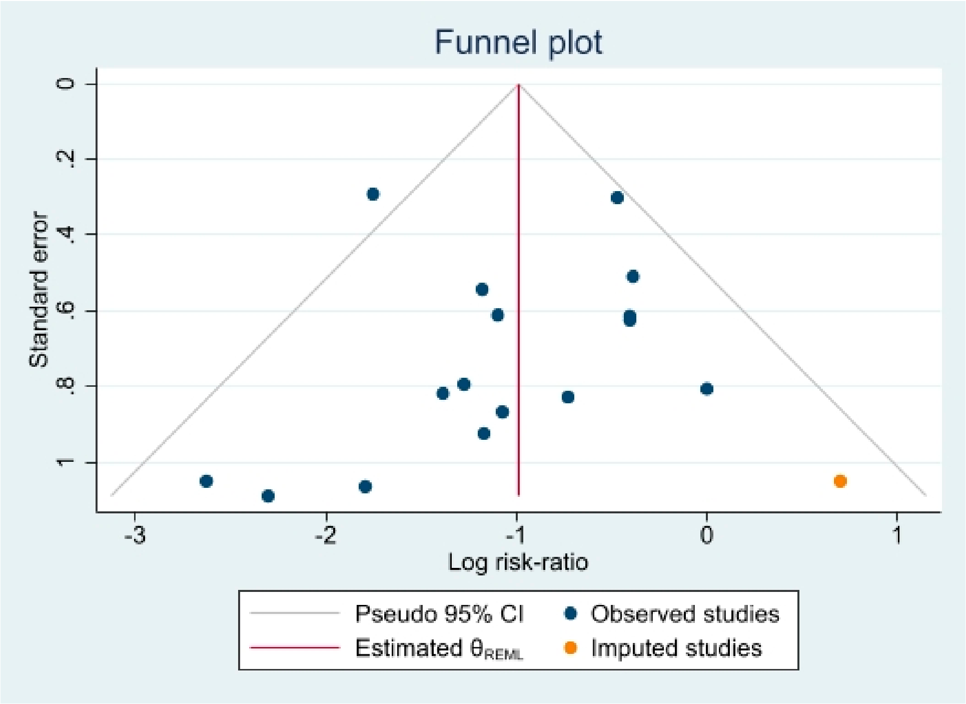

Supplementary Figure S2.3. Funnel plot with trim-and-fill method for the incidence of bradycardia. CI, confidence interval.

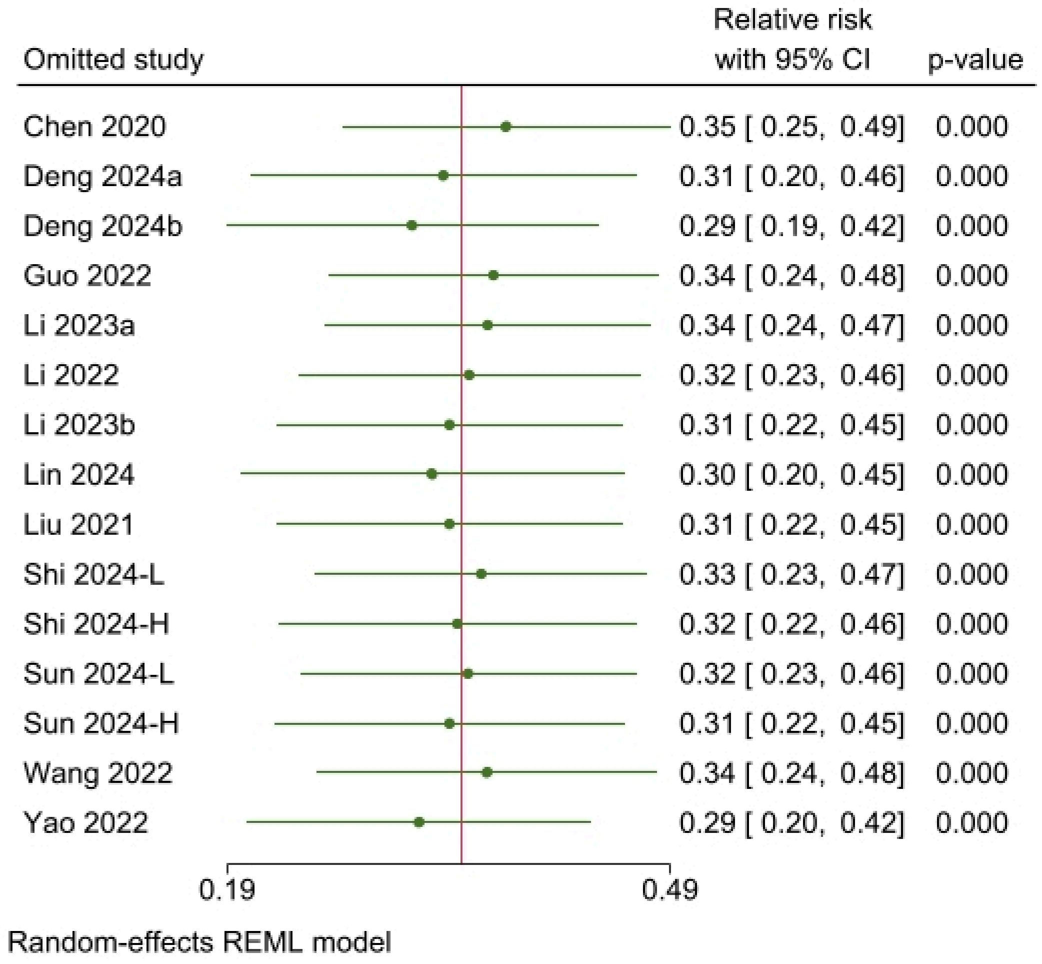

Supplementary Figure S3.1. Forest plot for sensitivity analysis of incidence of respiratory depression. The sensitivity analysis did not show any change in the significance of the pooled effect size. CI: confidence interval.

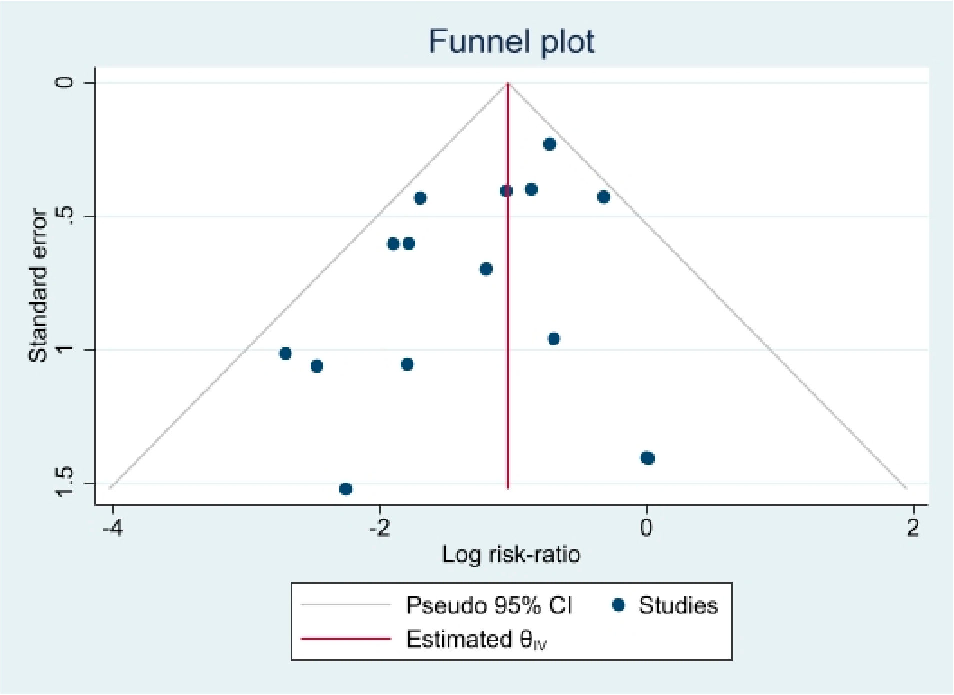

Supplementary Figure S3.2. Funnel plot for the incidence of respiratory depression. CI, confidence interval.

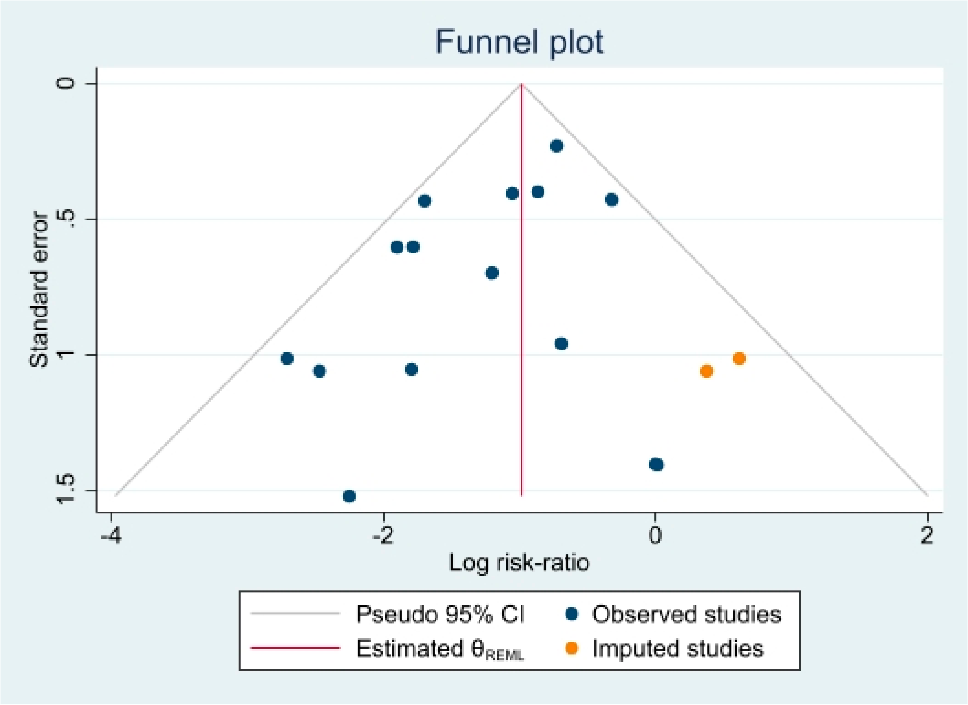

Supplementary Figure S3.3. Funnel plot with trim-and-fill method for the incidence of respiratory depression. CI, confidence interval.

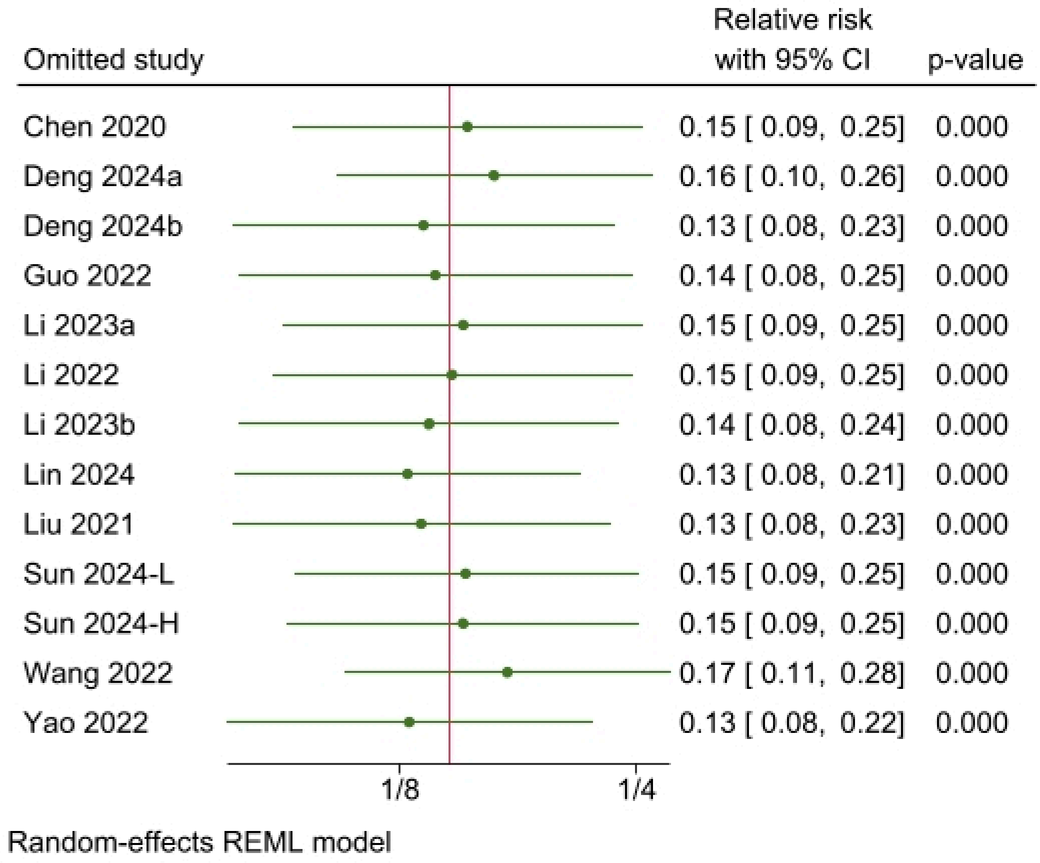

Supplementary Figure S4.1. Forest plot for sensitivity analysis of the incidence of injection pain. No meaningful changes were observed in the effect size. CI: confidence interval.

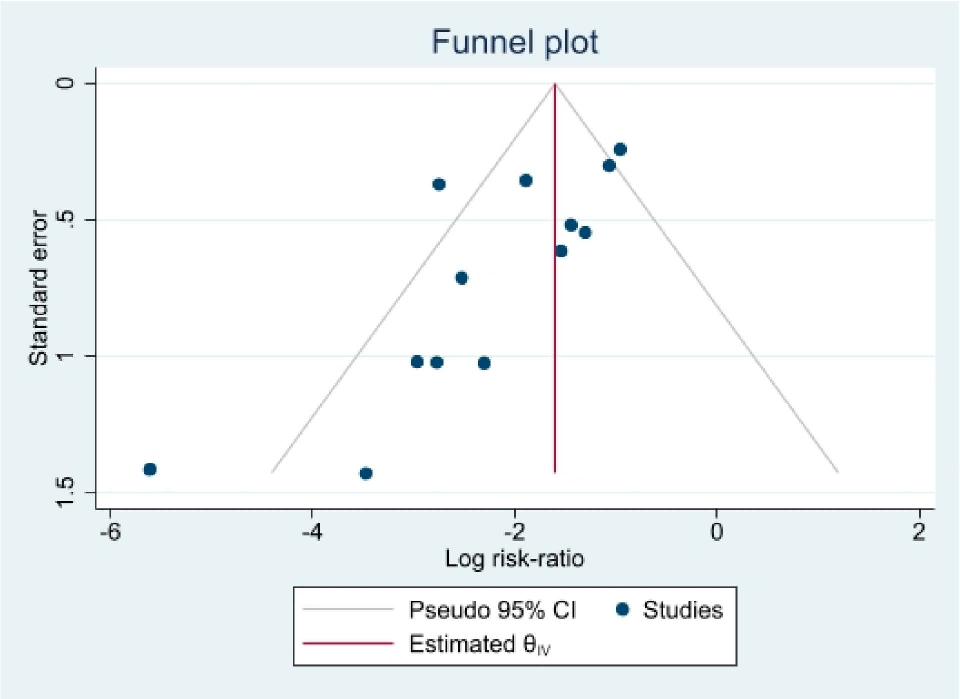

Supplementary Figure S4.2. Funnel plot for the incidence of injection pain. CI, confidence interval.

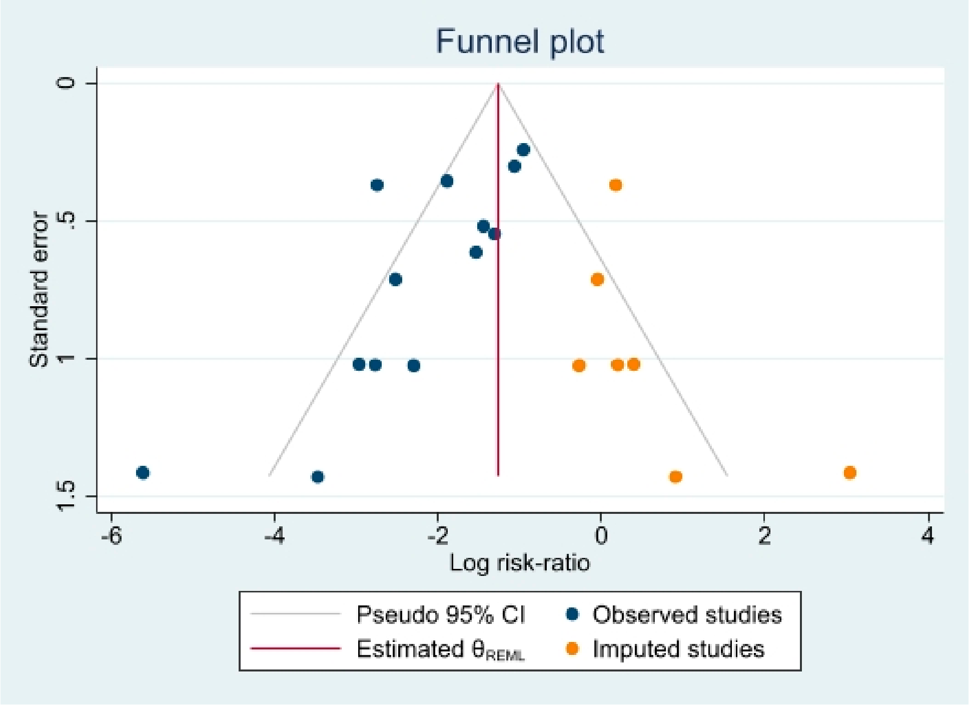

Supplementary Figure S4.3. Funnel plot with trim-and-fill method for the incidence of injection pain. CI, confidence interval.

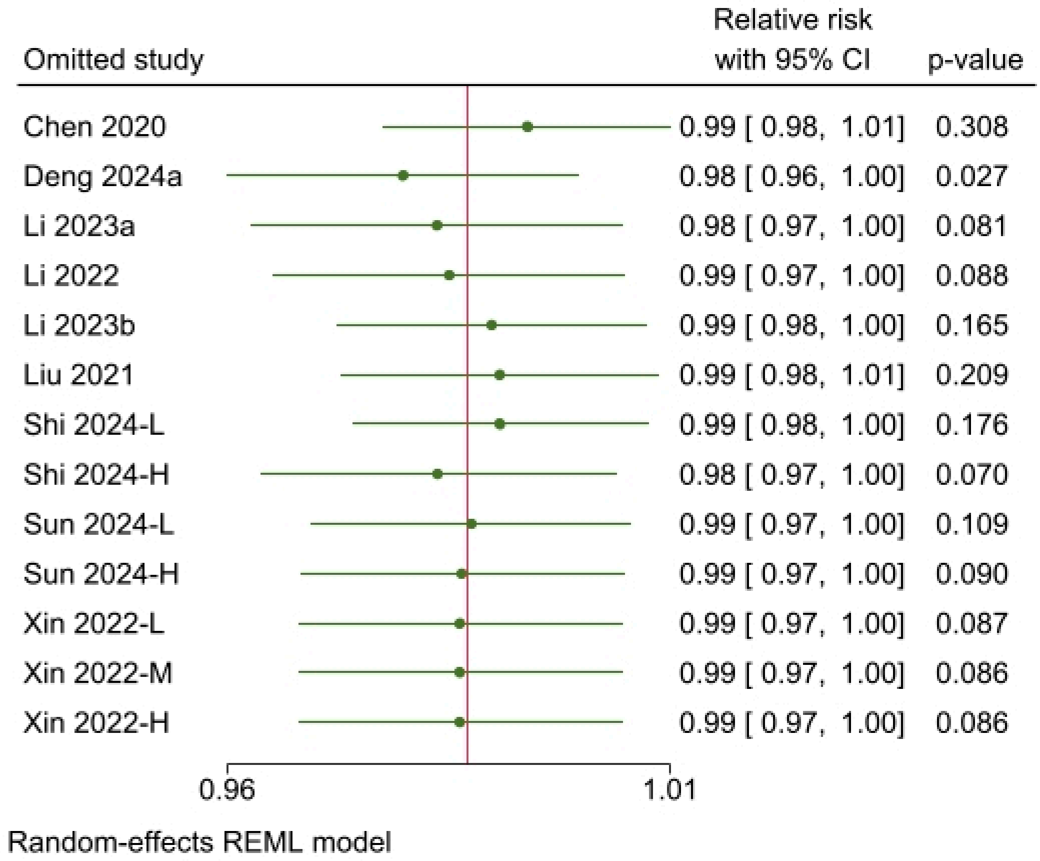

Supplementary Figure S5.1. Forest plot for sensitivity analysis of success rates. The sensitivity analysis revealed a change in the effect size upon omitting one study (Deng, 2024a). CI: confidence interval.

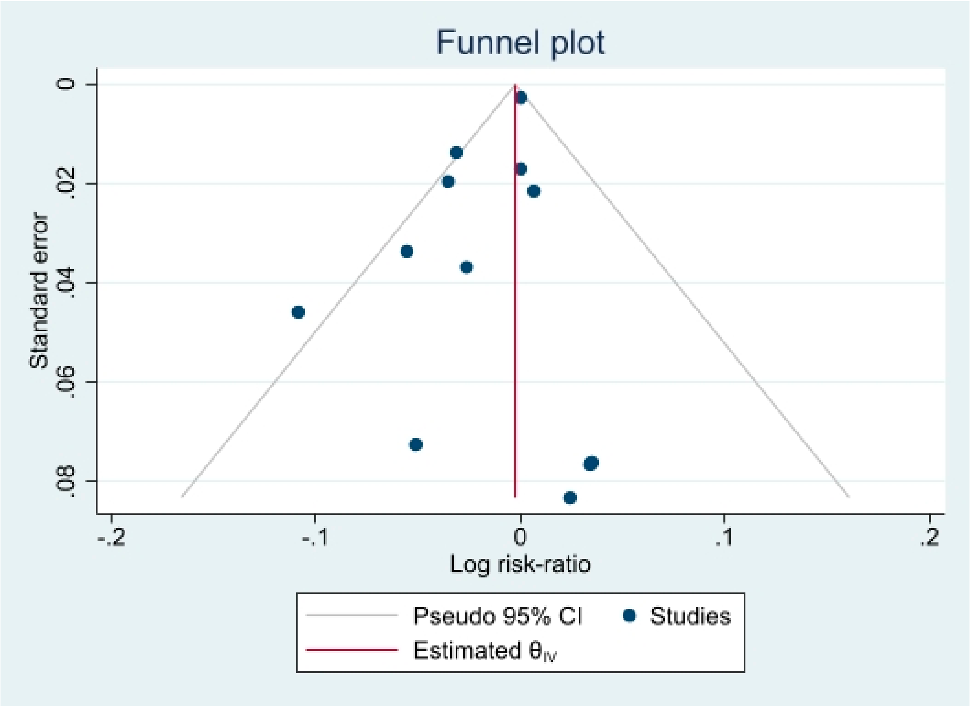

Supplementary Figure S5.2. Funnel plot for the success rates. CI, confidence interval.

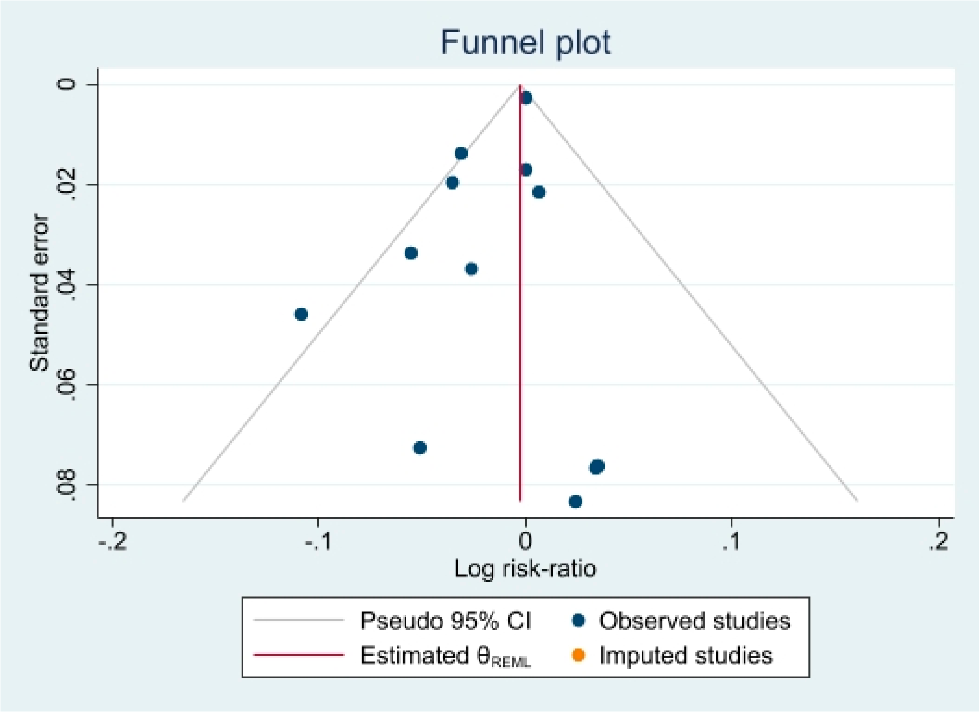

Supplementary Figure S5.3. Funnel plot with trim-and-fill method for the success rates. CI, confidence interval.

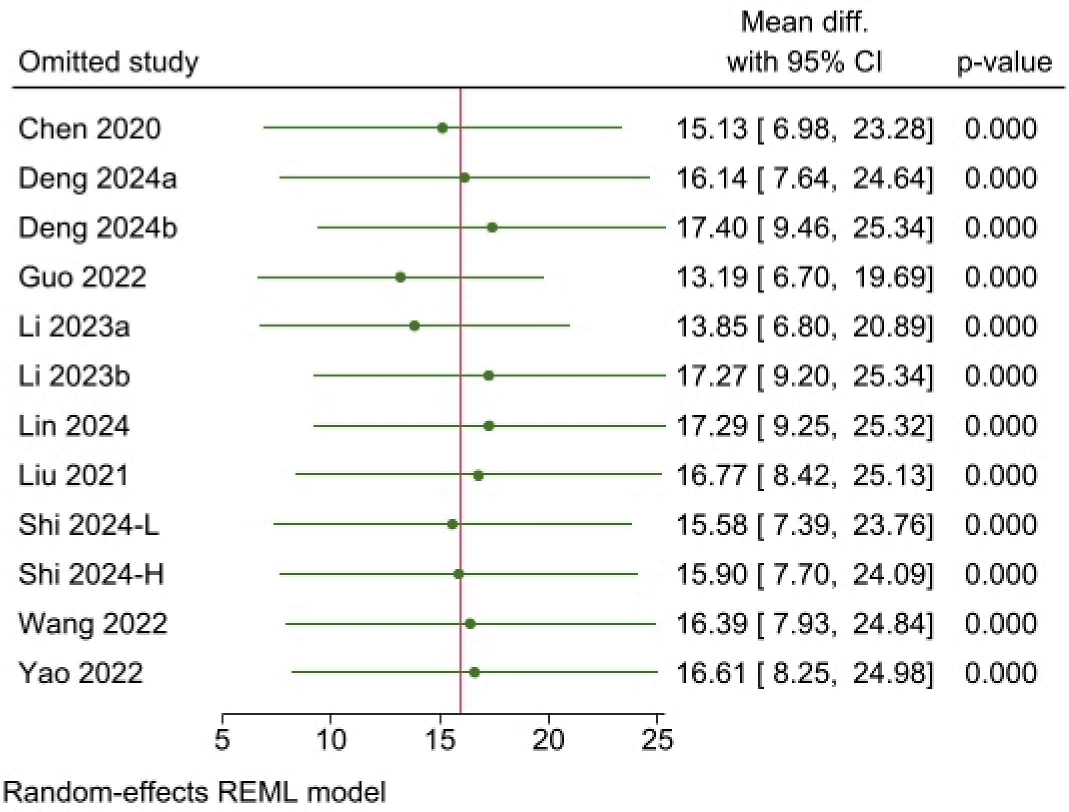

Supplementary Figure S6.1. Forest plot for sensitivity analysis of time to target sedation depth. Sensitivity analysis showed no effect size changes by omitting studies. CI: confidence interval.

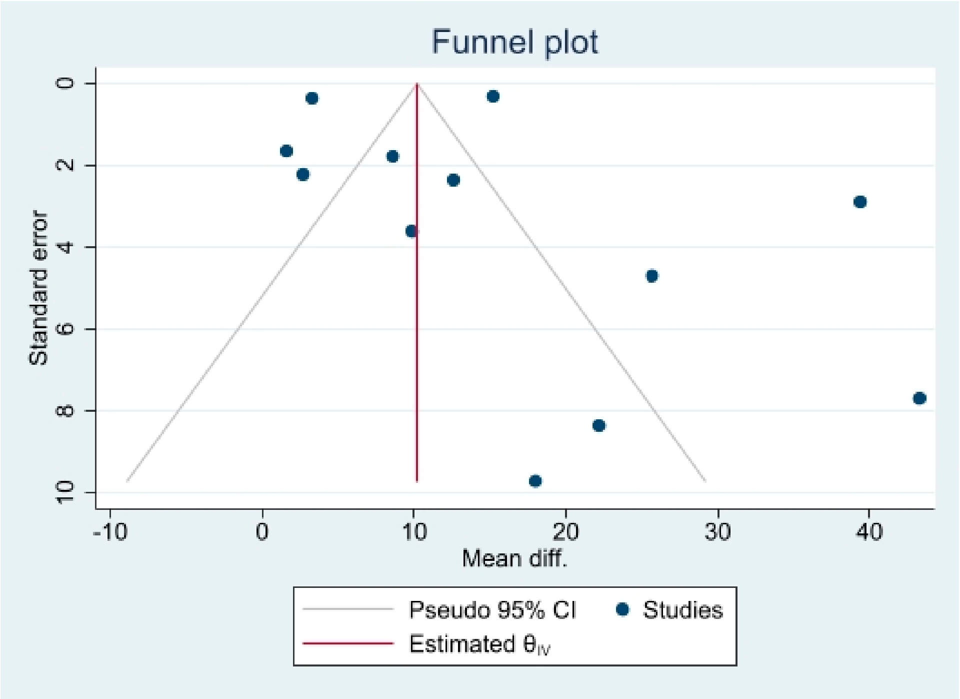

Supplementary Figure S6.2. Funnel plot for time to target sedation depth. CI, confidence interval.

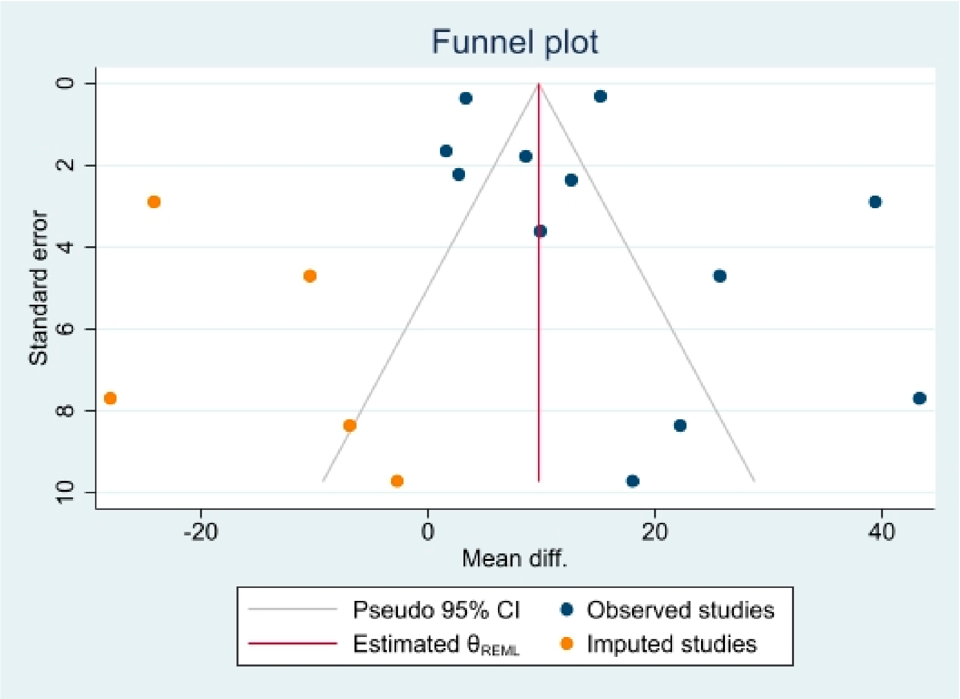

Supplementary Figure S6.3. Funnel plot with trim-and-fill method for time to target sedation depth. CI, confidence interval.

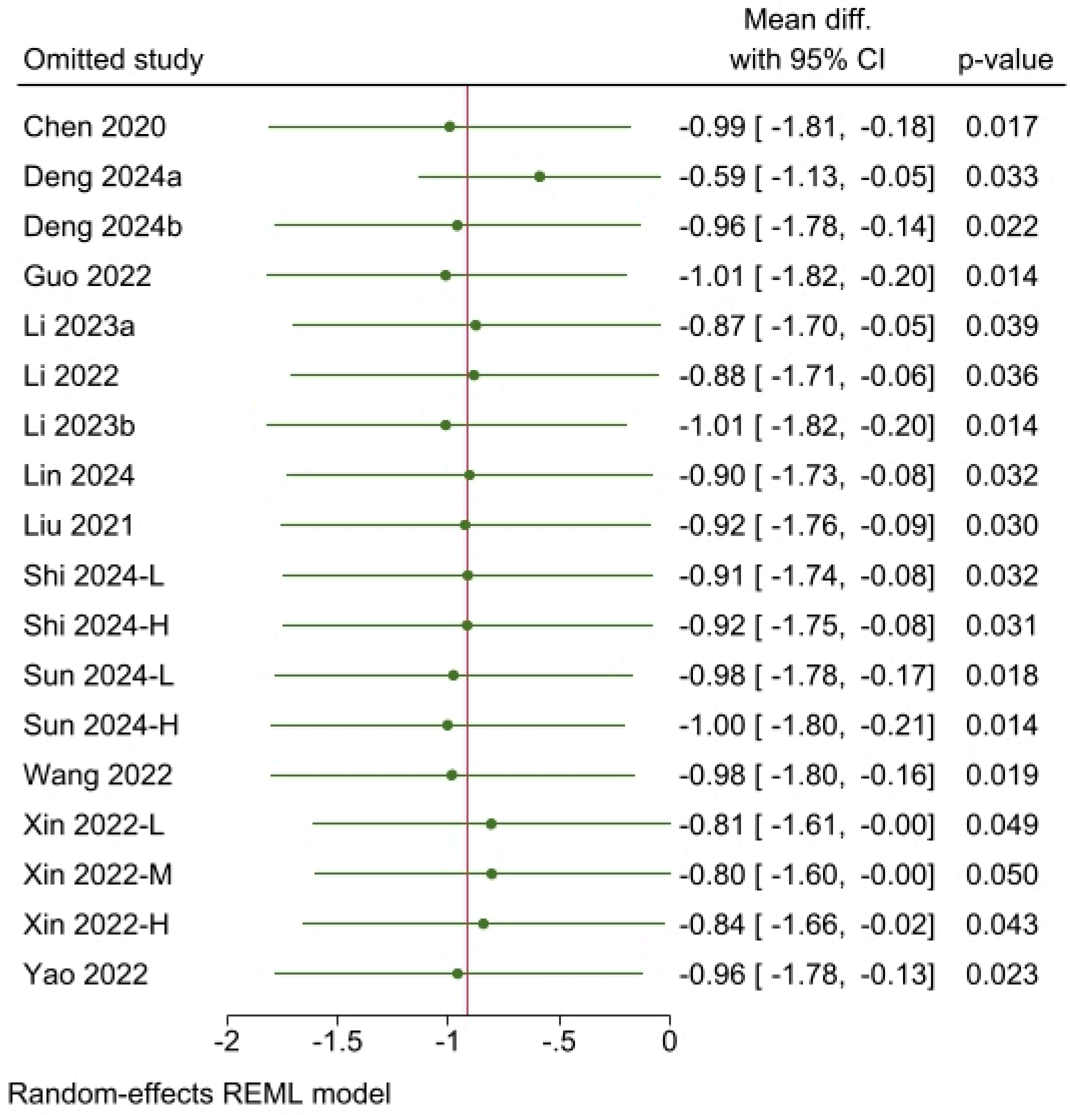

Supplementary Figure S7.1. Forest plot for sensitivity analysis of emergence time from sedation. No meaningful changes were observed in the effect size. CI: confidence interval.

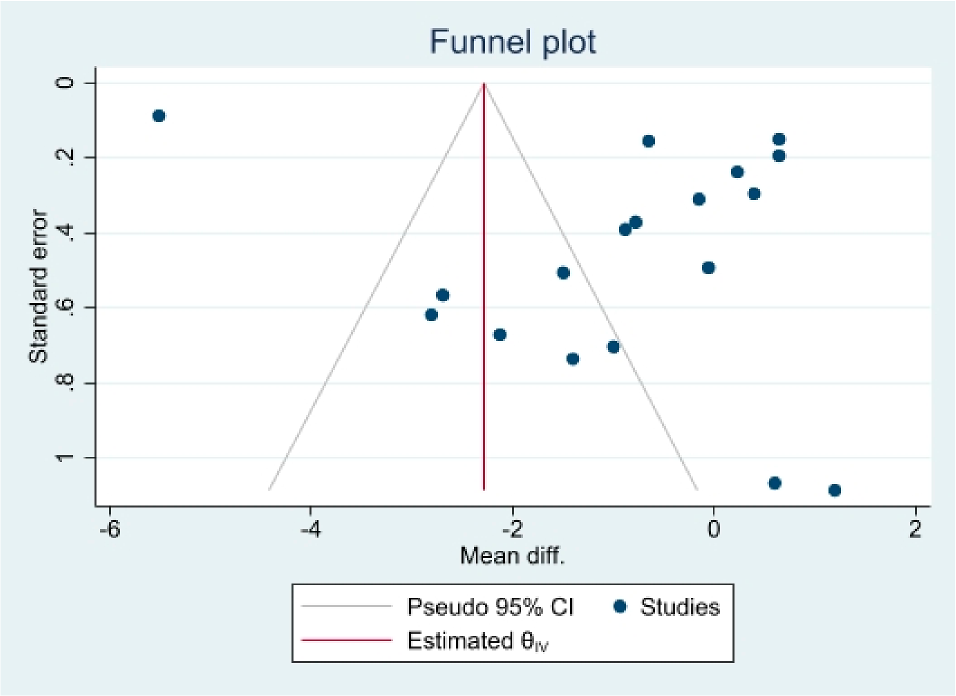

Supplementary Figure S7.2. Funnel plot for emergence time from sedation. CI, confidence interval.

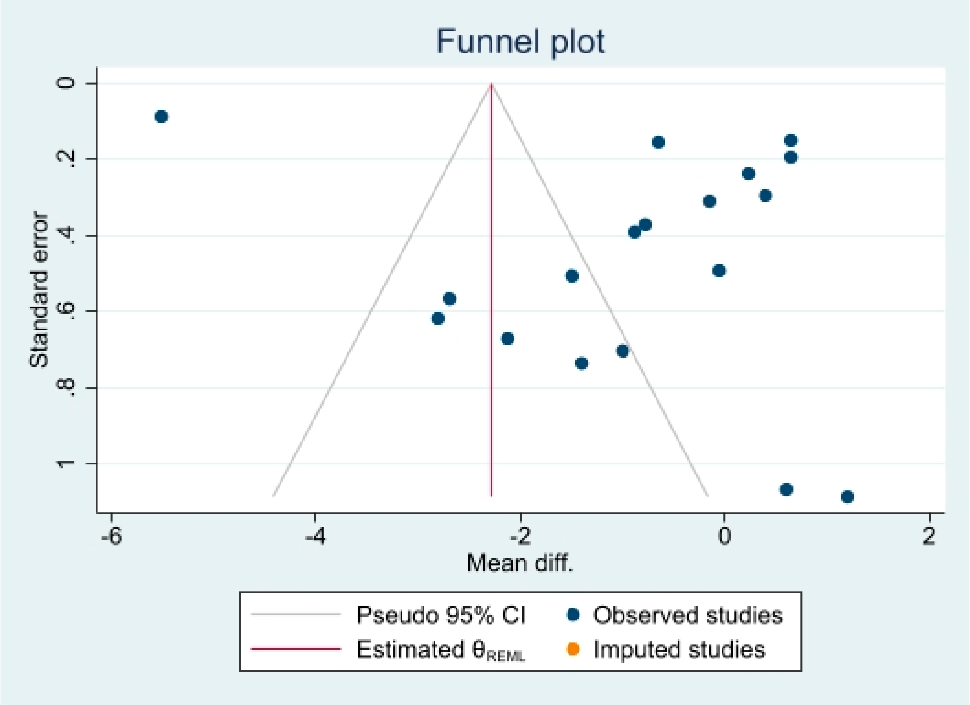

Supplementary Figure S7.3. Funnel plot with trim-and-fill method for emergence time from sedation. CI, confidence interval.

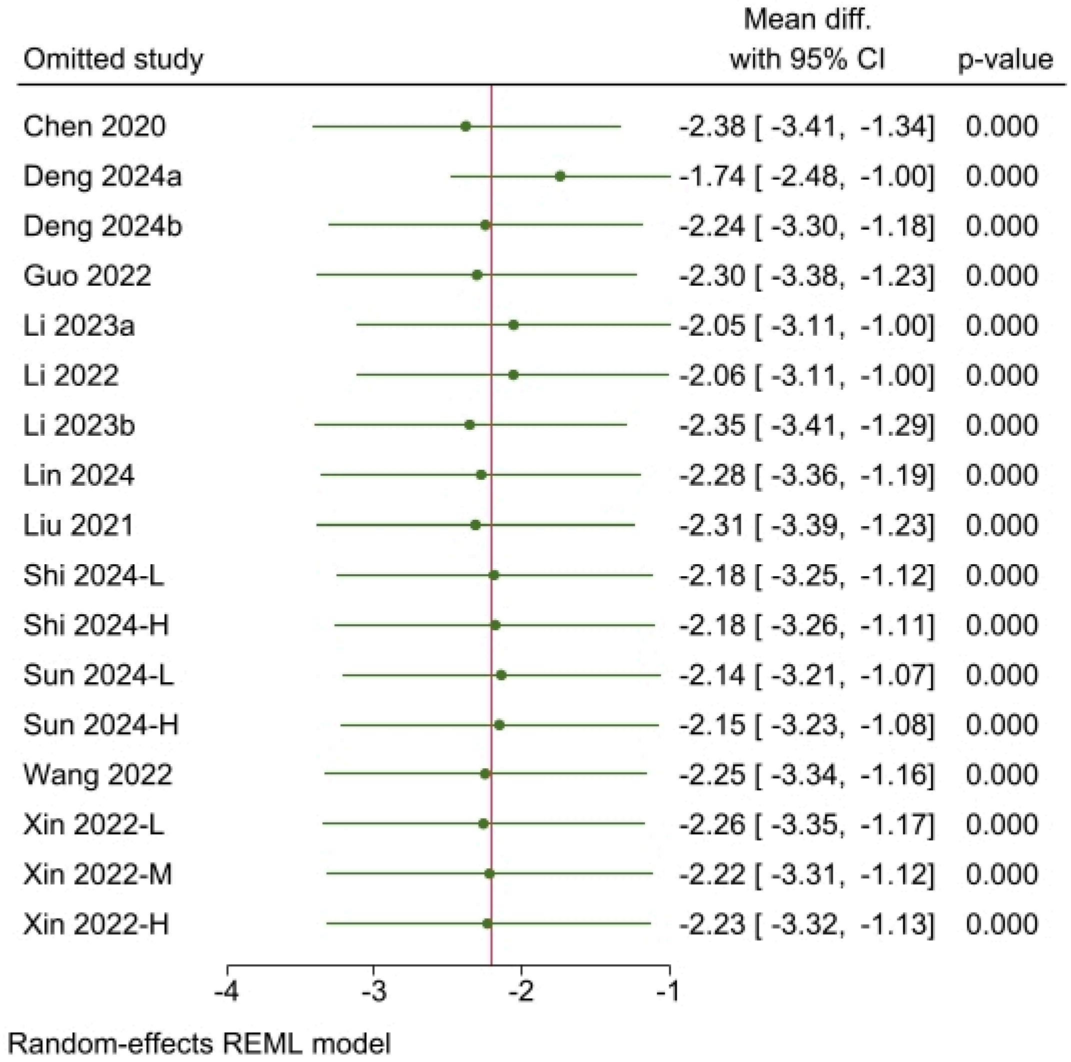

Supplementary Figure S8.1. Forest plot for sensitivity analysis of post-procedural unit stay time. Sensitivity analysis showed no effect size changes by omitting studies. CI: confidence interval.

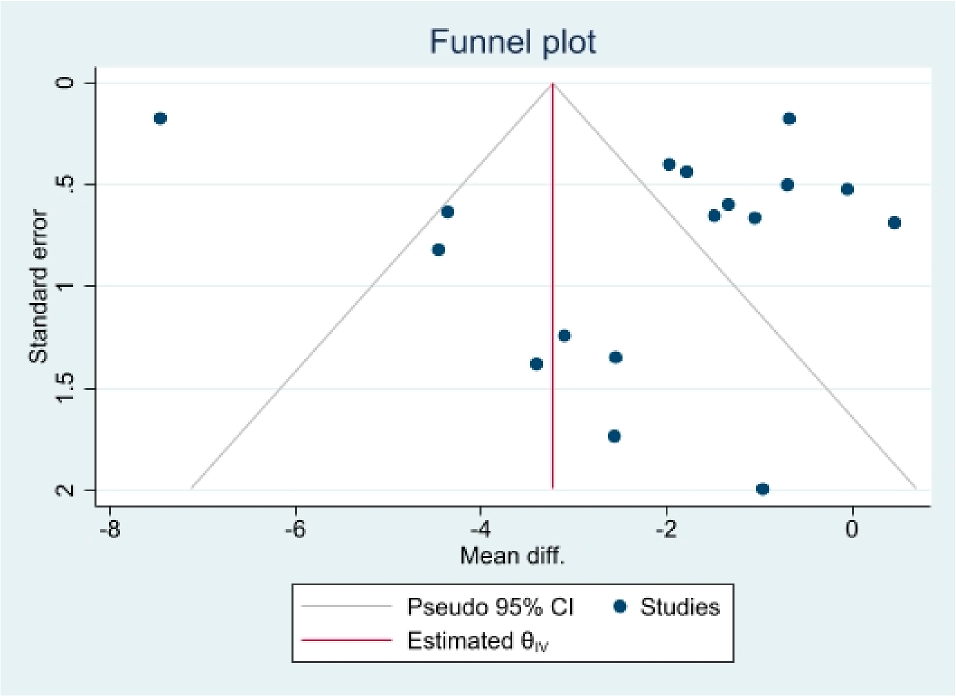

Supplementary Figure S8.2. Funnel plot for post-procedural unit stay time. CI, confidence interval.

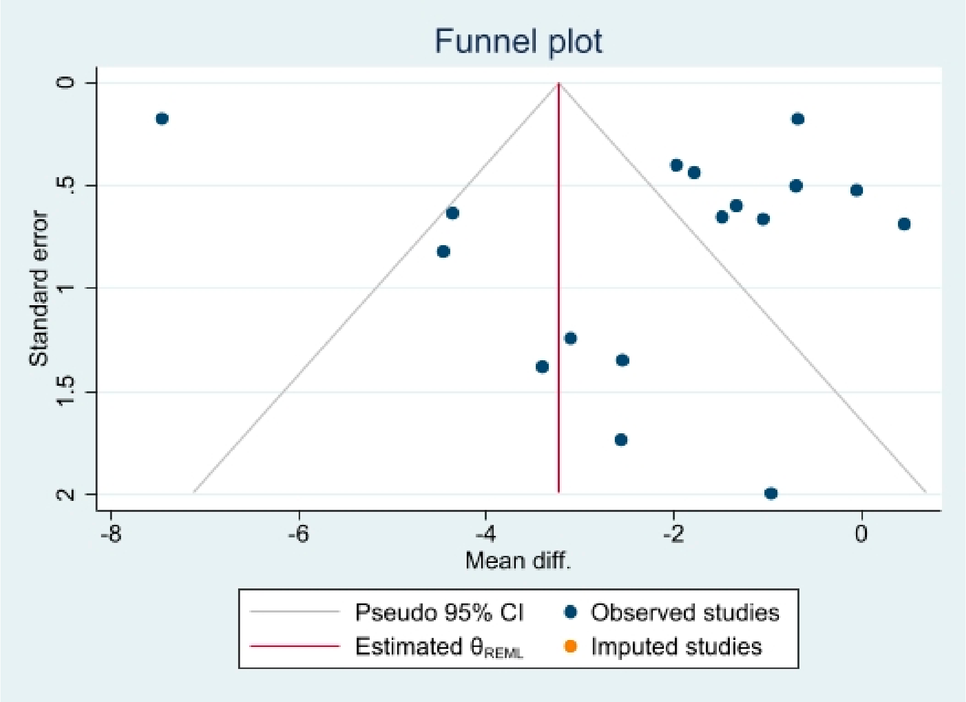

Supplementary Figure S8.3. Funnel plot with trim-and-fill method for post-procedural unit stay time. CI, confidence interval.

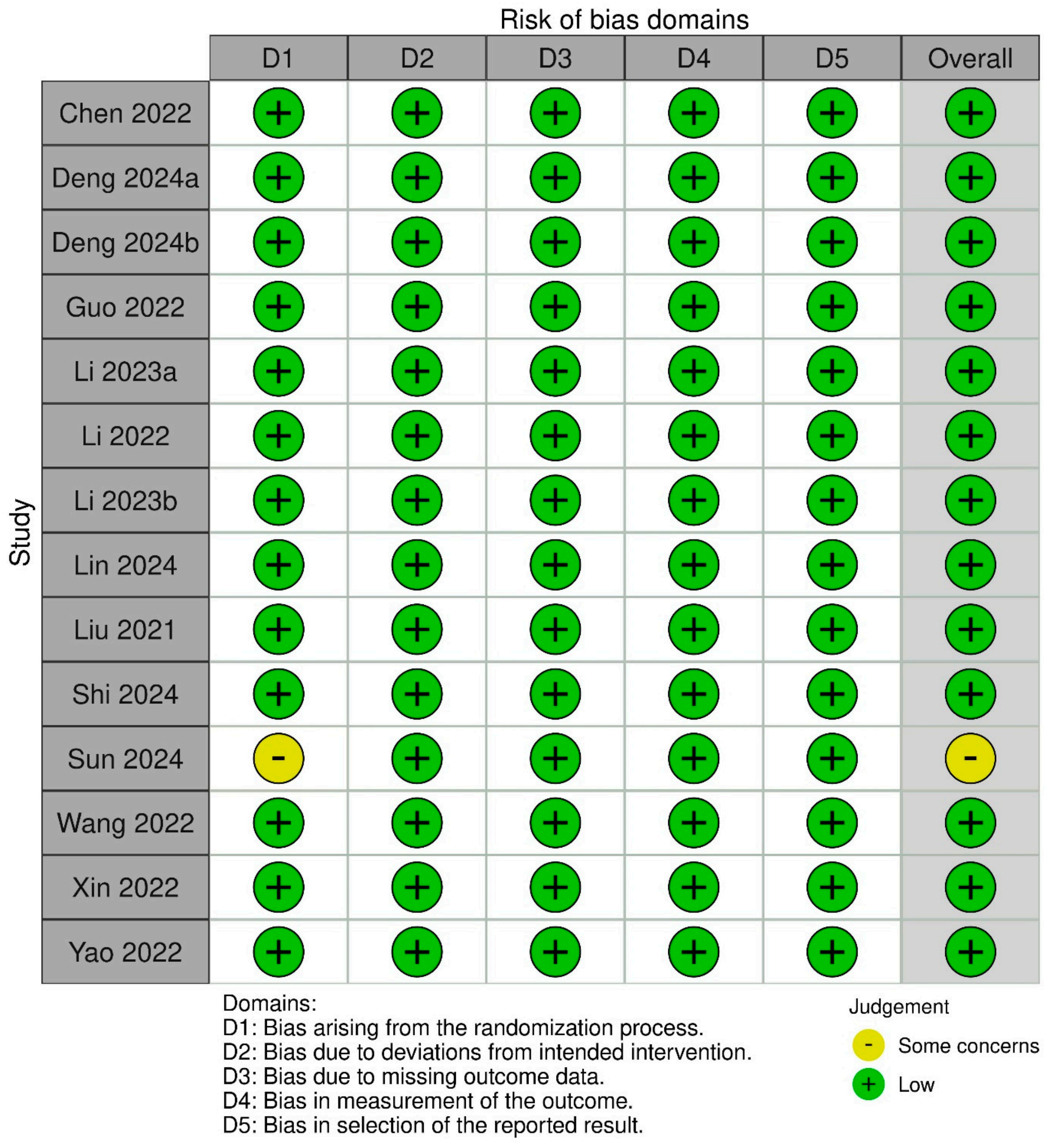

Supplementary Figure S9.1. Risk of bias summary. Green circle, low risk; yellow circle, some concerns; red circle, high risk

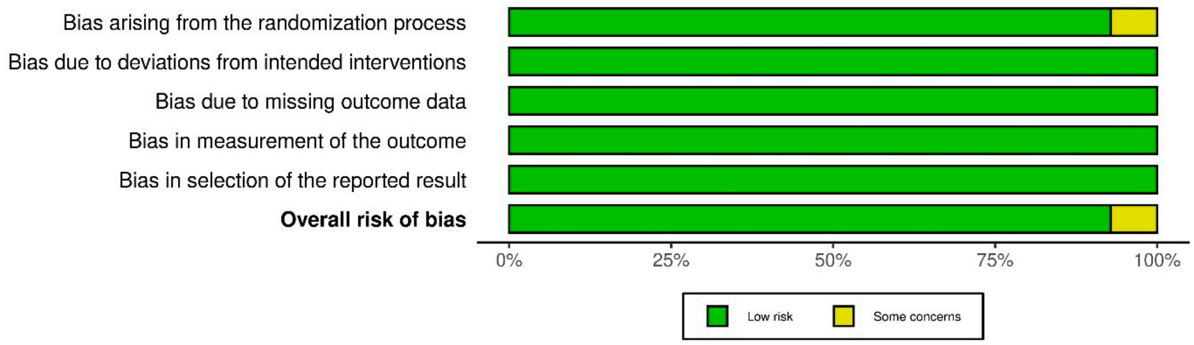

Supplementary Figure S9.2. Overall risk of bias as a summary plot.
